# LPIAT1/MBOAT7 depletion increases triglyceride synthesis fueled by high phosphatidylinositol turnover

**DOI:** 10.1136/gutjnl-2020-320646

**Published:** 2020-04-06

**Authors:** Yuki Tanaka, Yuta Shimanaka, Andrea Caddeo, Takuya Kubo, Yanli Mao, Tetsuya Kubota, Naoto Kubota, Toshimasa Yamauchi, Rosellina Margherita Mancina, Guido Baselli, Panu Luukkonen, Jussi Pihlajamäki, Hannele Yki-Järvinen, Luca Valenti, Hiroyuki Arai, Stefano Romeo, Nozomu Kono

**Affiliations:** 1 Department of Health Chemistry, Graduate School of Pharmaceutical Sciences, The University of Tokyo, Tokyo, Japan; 2 Department of Molecular and Clinical Medicine, Sahlgrenska Academy, University of Gothenburg, Gothenburg, Sweden; 3 Division of Diabetes and Metabolism, The Institute for Adult Diseases, Asahi Life Foundation, Tokyo, Japan; 4 Department of Diabetes and Metabolic Diseases, Graduate School of Medicine, The University of Tokyo, Tokyo, Japan; 5 Department of Clinical Nutrition Therapy, The University of Tokyo Hospital, The University of Tokyo, Tokyo, Japan; 6 Department of Pathophysiology and Transplantation, Università degli Studi di Milano, Milano, Italy; 7 Translational Medicine, Department of Transfusion Medicine and Hematology, Fondazione IRCCS Ca' Granda Ospedale Maggiore Policlinico, Milano, Italy; 8 Department of Medicine, University of Helsinki and Helsinki University Central Hospital, Helsinki, Finland; 9 Minerva Foundation Institute for Medical Research, Helsinki, Finland; 10 Department of Internal Medicine, Yale University, New Haven, CT, USA, Yale University, New Haven, Connecticut, USA; 11 Department of Clinical Nutrition, Institute of Public Health and Clinical Nutrition, University of Eastern Finland, Kuopio, Finland; 12 Clinical Nutrition and Obesity Center, Kuopio University Hospital, Kuopio, Finland; 13 AMED-CREST, Japan Agency for Medical Research and Development, Tokyo, Japan; 14 Present address: Laboratory of Microenvironmental and Metabolic Health Science, Center for Disease Biology and Integrative Medicine, Graduate School of Medicine, The University of Tokyo, Tokyo, Japan; 15 Clinical Nutrition Unit, Department of Medical and Surgical Science, Magna Graecia University, Catanzaro, Italy; 16 Department of Cardiology, Sahlgrenska University Hospital, Gothenburg, Sweden

**Keywords:** fatty liver, hepatic fibrosis, lipids, lipid metabolism

## Abstract

**Objective:**

Non-alcoholic fatty liver disease (NAFLD) is a common prelude to cirrhosis and hepatocellular carcinoma. The genetic *rs641738 C>T* variant in the lysophosphatidylinositol acyltransferase 1 (*LPIAT1)/membrane bound O-acyltransferase domain-containing 7*, which incorporates arachidonic acid into phosphatidylinositol (PI), is associated with the entire spectrum of NAFLD. In this study, we investigated the mechanism underlying this association in mice and cultured human hepatocytes.

**Design:**

We generated the hepatocyte-specific *Lpiat1* knockout mice to investigate the function of Lpiat1 in vivo. We also depleted *LPIAT1* in cultured human hepatic cells using CRISPR-Cas9 systems or siRNA. The effect of LPIAT1-depletion on liver fibrosis was examined in mice fed high fat diet and in liver spheroids. Lipid species were measured using liquid chromatography-electrospray ionisation mass spectrometry. Lipid metabolism was analysed using radiolabeled glycerol or fatty acids.

**Results:**

The hepatocyte-specific *Lpiat1* knockout mice developed hepatic steatosis spontaneously, and hepatic fibrosis on high fat diet feeding. Depletion of *LPIAT1* in cultured hepatic cells and in spheroids caused triglyceride accumulation and collagen deposition. The increase in hepatocyte fat content was due to a higher triglyceride synthesis fueled by a non-canonical pathway. Indeed, reduction in the PI acyl chain remodelling caused a high PI turnover, by stimulating at the same time PI synthesis and breakdown. The degradation of PI was mediated by a phospholipase C, which produces diacylglycerol, a precursor of triglyceride.

**Conclusion:**

We found a novel pathway fueling triglyceride synthesis in hepatocytes, by a direct metabolic flow of PI into triglycerides. Our findings provide an insight into the pathogenesis and therapeutics of NAFLD.

**Significance of this study**

**What is already known on this subject?**

- Non-alcoholic fatty liver disease (NAFLD), the most common chronic liver disease worldwide, is characterised by an increase in hepatic synthesis of triglycerides classically driven by carbohydrate.
- The genetic *rs641738 C>T* variant in the *membrane bound O-acyltransferase domain-containing 7* (*MBOAT7)/lysophosphatidylinositol acyltransferase 1 (LPIAT1)* associated with the entire spectrum of NAFLD. However, the mechanism underlying this association remains unclear.

**What are the new findings?**

- The hepatocyte-specific *Lpiat1* knockout mice developed steatosis and *LPIAT1* depletion in cultured human hepatocytes upregulates triglyceride synthesis.
- *LPIAT1* depletion in human and murine hepatocytes caused a high phosphatidylinositol (PI) turnover, which continuously produced diacylglycerol, a substrate for triglyceride synthesis.
- *LPIAT1* depletion exacerbated the fibrotic phenotype in liver spheroids and in mice fed high fat diet, recapitulating the human phenotype.

**How might it impact on clinical practice in the foreseeable future?**

- Classically, increased hepatic fat content may be due to higher de novo lipogenesis due to insulin action or high fructose intake. In this work, we show a novel pathway where lipogenesis is due to a direct metabolic flow of PI into triglycerides caused by downregulation of *MBOAT7/LPIAT1*. Ultimately, this novel de novo lipogenesis pathway may be used as therapeutic target to treat NAFLD.

## Introduction

In parallel with the obesity epidemics, non-alcoholic fatty liver disease (NAFLD) is an emerging threat for human health. NAFLD comprises a spectrum of conditions, going from excess in hepatic triglyceride (TG) content in the absence of alcohol consumption, leading to cirrhosis and hepatocellular carcinoma. Increase in TG synthesis is one of the mechanisms leading to NAFLD.^1 2^ Lipogenesis may be mediated by at least two mechanism: (1) a high flux of carbon down glycolysis (as during excess in fructose intake^3 4^), supplying excess triose phosphates, which serve as the glycerol backbone for TG synthesis; (2) a sterol regulatory element-binding protein 1c (SREBP1c)-mediated production of fatty acids due to fatty acid synthesis.^2 5^ NAFLD has also a strong genetic susceptibility, and among the genes of susceptibility^6^ the single nucleotide polymorphism (*rs641738 C>T*) in the *membrane bound O-acyltransferase domain-containing 7 (MBOAT7*) gene increases the susceptibility to the entire spectrum of NAFLD,^7 8^ by inducing a reduction in the liver *MBOAT7* mRNA expression.^8–11^

*MBOAT7* encodes lysophosphatidylinositol acyltransferase 1 (LPIAT1), which preferentially incorporates arachidonic acid (AA; 20:4 n-6) into phosphatidylinositol (PI).^12–14^ PI is a constitutive of membrane phospholipids and the precursor of signalling lipid phosphoinositides,^15^ but so far no direct role of PI in TG synthesis has been shown.

Unlike other major membrane phospholipids containing various unsaturated fatty acids, PI contains most exclusively AA as acyl chain in the *sn*-2 position.^16 17^ Individuals homozygous for rare disruptive mutations in the *LPIAT1/MBOAT7* gene (hereafter called *LPIAT1*) have severe intellectual disability, epilepsy and autistic features.^18^ Consistently, *Lpiat1*-deficient mice exhibits aberrant brain development accompanied by disordered lamination and neuronal processes in the cortex, suggesting that AA-containing PI produced by LPIAT1 is essential for neonatal neural development.^13^ However, as *Lpiat1*-deficient mice die within a month after birth, the mechanism by which *Lpiat1* deficiency causes liver steatosis has not been investigated.

## Results

### Depletion of *Lpiat1* in adult mice causes steatosis

**Figure 1:**
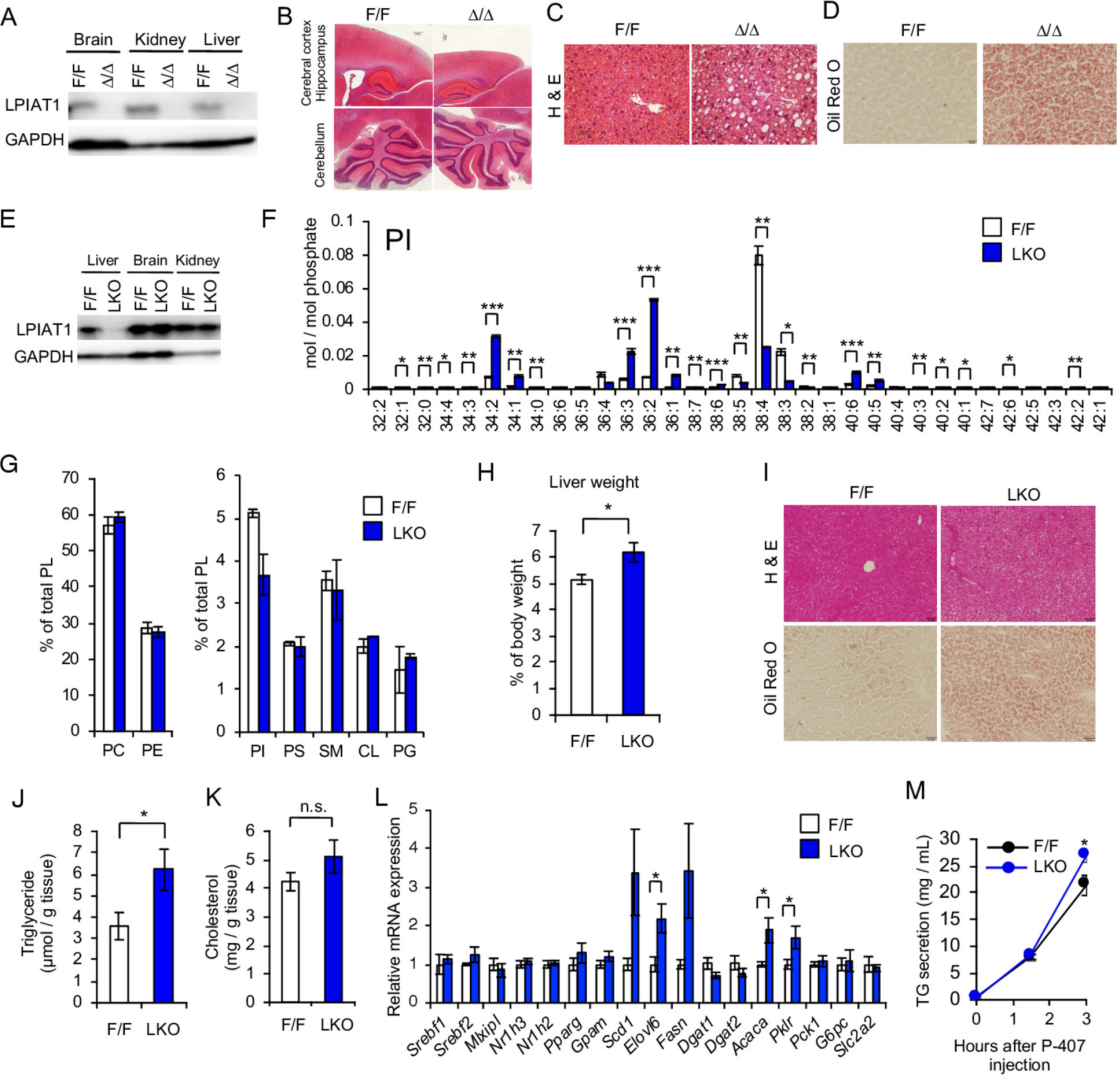
Hepatocyte-Specific LPIAT1 depletion causes steatosis. (A–D) Six-week-old male *Lpiat1^f/f^* mice (F/F) and *Ubc-CreER^T2^;Lpiat1^f/f^* mice (∆/∆) were treated with tamoxifen for 3 weeks. (A) Western blot of LPIAT1 in brain, kidney and liver. GAPDH serves as a control. Similar results were obtained in one additional independent experiment. (B–D) Representative images of brain (B) or liver (C, D) sections from tamoxifen-treated F/F and ∆/∆ mice stained with H&E (B, C) or Oil-red O (D). Images are representative of three independent experiments. (E) Western blot of LPIAT1 in liver, brain and kidney from 12-week-old male F/F and hepatocyte-specific *Lpiat1* knockout (LKO) mice. GAPDH serves as a control. Similar results were obtained in one additional independent experiment. (F) LC-MS/MS analysis of phosphatidylinositol (PI) species in the liver of F/F and LKO mice (n=3). (G) The content of individual phospholipid of the livers from F/F and LKO mice. (H) Liver weights of F/F and LKO mice normalised to the total body weight (n=4). (I) Representative images of the liver sections from F/F and LKO stained with H&E (upper) and oil red O (below). Images are representative of three independent experiments. (J, K) Levels of hepatic triglyceride (n=6) (J) and cholesterol (n=4) (K) in F/F and LKO mice. (L) Expression of genes involved in lipogenesis, β-oxidation and gluconeogenesis in the liver of F/F and LKO mice (n=4–6). (M) Measurement of triglyceride (TG) secretion rate. Lipoprotein lipase inhibitor (P-407, 1 g / kg body weight) was intraperitoneally injected and serum TG levels were measured at each time point (n=5). Values are shown as mean±SEM data were analysed by unpaired two-tailed Student’s t-test (F, H, J, K, L, M): *p<0.05, **p<0.01, ***p<0.001. CL, cardiolipin; GAPDH, glyceraldehyde 3-phosphate dehydrogenase; H&E, hematoxylin and eosin; LC-MS/MS, liquid chromatography-mass spectrometry; LKO, hepatocyte-specific Lpiat1 knockout; LPIAT1, lysophosphatidylinositol acyltransferase 1; n.s., not significant; PC, phosphatidylcholine; PE, phosphatidylethanolamine; PG, phosphatidylglycerol; PL, phospholipid; PS, phosphatidylserine; SM, sphingomyelin.

To bypass the neonatal lethality of *Lpiat1*-deficient mice, we first generated the tamoxifen-inducible *Lpiat1* knockout mice (*Ubc-CreER^T2^; Lpiat1^f/f^*), by mating *Lpiat1*-floxed mice (*Lpiat1^f/f^*) with the transgenic mice carrying tamoxifen-inducible Cre transgene (*Ubc-CreER^T2^*)^19^ (online supplementary figure 1A). In *Ubc-CreER^T2^; Lpiat1^f/f^* mice, *Lpiat1^f^* alleles were almost completely converted to deleted alleles (*Lpiat1^Δ^*) within 1 week after tamoxifen treatment (online supplementary figure 1B). LPIAT1 protein measured by western blotting was absent in all of the tissues examined, including brain, kidney and liver, after 2 weeks after tamoxifen treatment (figure 1A, online supplementary figure 1C). Tamoxifen-treated *Ubc-CreER^T2^;Lpiat1^f/f^* (*Lpiat1^Δ/Δ^*) mice fed on chow diet grew normally for at least 2 months. As expected, liquid chromatography-electrospray ionisation mass spectrometry (LC/ESI-MS) analysis showed that the amount of 38:4 PI (most probably 18:0/20:4 PI) was dramatically reduced in *Lpiat1^Δ/Δ^* liver, whereas other PI species, such as 34:1 and 36:2, were slightly increased (online supplementary figure 1D). In contrast, molecular species of phosphatidylcholine (PC) in *Lpiat1^Δ/Δ^* liver were similar to those in *Lpiat1^f/f^* liver (online supplementary figure 1E). Contrary to conventional *Lpiat1* knockout mice,^13^ *Lpiat1^Δ/Δ^* mice showed a histologically normal brain morphology (figure 1B). Consistently with the carriers of the *MBOAT7 rs641738 C>T* variant, *Lpiat1^Δ/Δ^* livers showed accumulation of neutral lipids (figure 1C, D, online supplementary figure 1F). These results suggest that the depletion of *Lpiat1* in adult mice results in an increased hepatic TG content.

10.1136/gutjnl-2020-320646.supp1 Supplementary data

### Hepatocyte-specific *Lpiat1*-deficient mice develop steatosis

Next, to examine whether *Lpiat1* specific depletion in hepatocytes causes steatosis, we generated hepatocyte-specific *Lpiat1* knockout mice (LKO), by crossing the *Lpiat1^f/f^* mice with the albumin-Cre (*Alb-Cre*) mice.^20^ LPIAT1 was depleted in the liver in LKO mice as shown by western blotting (figure 1E). Similarly to *Lpiat1^Δ/Δ^* liver, the amount of AA-containing PI (38:4 PI) was dramatically reduced in LKO liver, whereas other PI species, such as 34:2, 34:1, 36:3, 36:2 and 36:1 PI, were significantly increased (figure 1F). There was no accumulation of lysoPI, the major lipid substrate of LPIAT1, in LKO liver (online supplementary figure 2A). PI content was slightly decreased in LKO liver (figure 1G). There was not difference in the amount of other phospholipids (PC, phosphatidylethanolamine (PE), phosphatidylserine (PS), sphingomyelin (SM), cardiolipin (CL) and phosphatidylglycerol (PG)) (figure 1G, online supplementary figure 2B-E), except the 34:2 PG and 36:2 PG that were increased in LKO liver (online supplementary figure 2E). LKO mice displayed higher liver weights compared with control mice (*Lpiat1^f/f^*) (figure 1H). Hepatocyte-specific depletion of *Lpiat1* also caused significant increase in neutral liver fat content in the form of TGs, as revealed by Oil red O staining and biochemical measurement (figure 1I, J). There was no difference in hepatic cholesterol content between LKO and *Lpiat1^f/f^* liver (figure 1K). mRNA levels of several genes involved in lipogenesis, including *Srebf1*, were unchanged (figure 1L). mRNA levels of genes involved in fatty acid β-oxidation and gluconeogenesis were not significantly changed, except for *Pklr* that was increased in LKO mice (figure 1L). Taken all together, these results show that *Lpiat1* depletion in hepatocytes causes (1) remodelling of PI with an overall depletion of AA, and (2) increased liver TG content.

Next, to understand the mechanism underlying the increase in liver fat content, we then examined the rate of hepatic TG secretion in the form of very low-density lipoprotein (VLDL) in LKO mice. Fasted mice were injected intraperitoneally poloxamer 407, a lipoprotein lipase inhibitor^21^ and TG concentration in plasma were assessed. There was no decrease in the rate of hepatic TG secretion between LKO mice and *Lpiat1^f/f^* mice (figure 1M), indicating an absence of VLDL retention in the liver. However, total plasma TG was decreased to approximately half in LKO mice (online supplementary figure 2F), and VLDL-TG was reduced among the plasma lipoprotein fractions in LKO plasma (online supplementary figure 2G). This might be explained by an increase in the catabolism of VLDL-TG mediated by LPL and other lipases. The total plasma cholesterol levels and plasma cholesterol distribution among the plasma lipoproteins were comparable between LKO and *Lpiat1^f/f^* mice (online supplementary figure 2H, I).

### *LPIAT1* depletion causes cellular TG accumulation in cultured human hepatocytes

**Figure 2:**
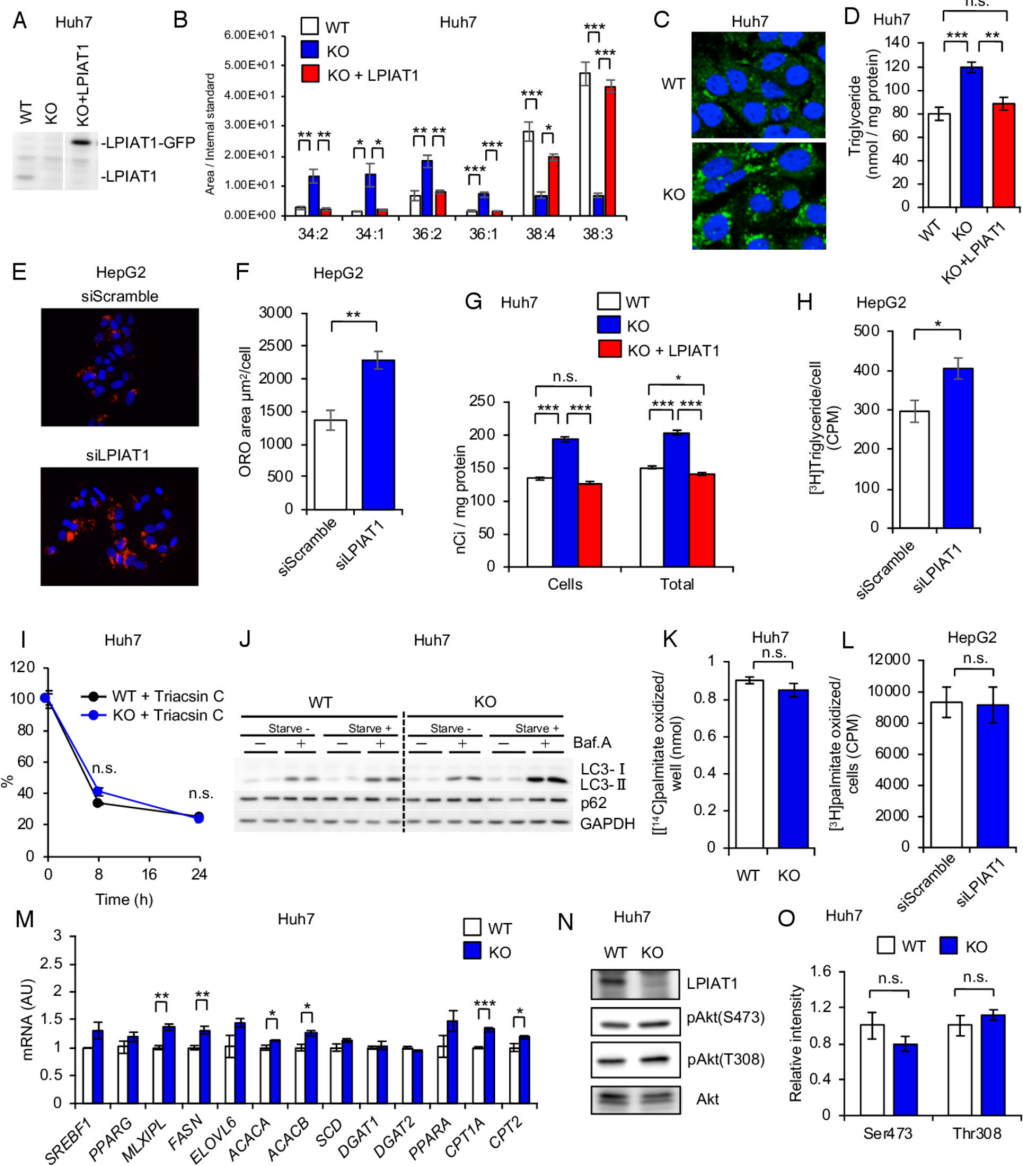
LPIAT1 depletion causes cellular TG accumulation in cultured hepatocytes (A) Western blot of LPIAT1 in native Huh-7 cells (WT), *LPIAT1* knockout Huh-7 cells (KO) and KO Huh-7 cells reconstituted with GFP-tagged LPIAT1 (KO+LPIAT1). GAPDH serves as a control. Similar results were obtained in one additional independent experiment. (B) LC-MS/MS analysis of PI species in WT, KO and KO+LPIAT1 Huh-7 cells (n=3). (C) Representative images of WT and KO Huh-7 cells stained with BODIPY. Similar results were obtained in one additional independent experiment. (D) Cellular TG levels of WT, KO and KO+LPIAT1 Huh-7 cells (n=4–5). (E, F) HepG2 cells were treated with scramble siRNA (siSramble) or LPIAT1 siRNA (siLPIAT1) and subjected to oil red O staining. Representative oil red O fluorescent staining images (E). Neutral lipid content quantified by ImageJ and normalised to number of nuclei (F; n=4). (G) Incorporation of [^14^C]oleic acid into TG. WT, KO and KO +LPIAT1 Huh-7 cells were incubated with [^14^C]oleic acid for 24 hours and both cellular and medium lipids were extracted and separated by TLC. Radioactivity in TG fractions was measured (n=4). (H) De novo synthesis of TG in HepG2 cells using [^3^H]glycerol (n=5). (I) Chase analysis of [^14^C]glycerol-labelled TG. Cells were preincubated with [^14^C]glycerol for 12 hours and then shifted to radioactivity free medium with triacsin C (20 µM) and radioactivity of TG fractions at each time point was measured. Data were shown by the relative ratio of signal intensity, with the value at 0 hour set as 100% (n=3). (J) Western blot of LC3 and p62 of WT and KO Huh-7 cells treated with or without EBSS and bafilomycin A1 (150 nM). GAPDH serves as a control. Similar results were obtained in one additional independent experiment. (K) Measurement of β-oxidation rate of WT and KO Huh-7 cells. After incubation with [^14^C]palmitic acid, radioactivity of acid-soluble fractions were measured by scintillation counting (n=4). (L) β-oxidation rate of HepG2 cells treated with siScramble or siLPIAT1. After incubation with [^3^H]palmitic acid, radioactivity of acid-soluble fractions were measured by scintillation counting (n=4). (M) Expression of genes involved in TG metabolism (n=3). (N, O) Western blot analysis of Akt phosphorylation in WT and KO Huh-7 cells (N). The ratios of p-Akt to Akt were mersured, with the value of WT being as 1 (O) (n=3). Values are shown as mean±SEM data were analysed by one-way ANOVA with Tukey’s post hoc test (B, D, G) or unpaired two-tailed Student’s t-test (F, H, I, K, L, M, O): *p<0.05, **p<0.01, ***p<0.001. ANOVA, analysis of variance; GAPDH, glyceraldehyde 3-phosphate dehydrogenase; HepG2, hepatoma cell line; Huh-7, hepatoma cell line; KO, knockout; LC-MS/MS, liquid chromatography-mass spectrometry; LPIAT1, lysophosphatidylinositol acyltransferase 1; n.s., not significant; PI, phosphatidylinositol; TG, triglyceride; TLC, thin layer chromatography; WT, wild type.

To elucidate the molecular mechanism causing steatosis in LKO mice, we generated hepatoma cell line (Huh-7) deficient in *LPIAT1* using CRISPR-Cas9 system (figure 2A). In Huh-7 cells, a substantial amount of 20:3-containing PI (38:3 PI) was detected. This is probably because Mead acid (20:3) is synthesised from oleic acid as a substitute of 20:4 in the cells cultured with foetal bovine serum (FBS)-containing medium, which contains much less essential fatty acids than murine plasma and tissues.^22^ Like in LKO liver, the amount of 20:4- and 20:3- containing PI (38:4 and 38:3 PI) were dramatically reduced in *LPIAT1* knockout (KO) Huh-7 cells, while other PI species, such as 34:2, 34:1, 36:2 and 36:1 PI, were increased (figure 2B). Amounts of other phospholipid classes were not different except for the amounts of PG (especially 34:2 and 36:2 PG) that were increased in *LPIAT1* KO Huh-7 cells (online supplementary figure 3A-E).

*LPIAT1* KO Huh-7 cells showed higher intracellular levels of TGs, as revealed by boron-dipyrromethene (BODIPY) staining and biochemical measurement, without affecting the fatty acid composition of TG (figure 2C, D, online supplementary figure 3F). Retroviral expression of GFP-tagged LPIAT1 completely rescued the composition of PI species and TG content in *LPIAT1* KO Huh-7 cells (figure 2A, B, D). To confirm our data in a different human hepatic cell line, we downregulated *LPIAT1* in HepG2 cells, by using siRNAs and measured intracellular neutral lipid content by fluorescent Oil Red O staining (figure 2E, F). Consistently with our data in Huh-7 cells, *LPIAT1* downregulation results in a twofold increase in intracellular lipid content (figure 2E, F). Taken all together, these data demonstrate that *LPIAT1* deficiency causes intracellular TG accumulation in a hepatocyte-autonomous manner.

### Depletion of *LPIAT1* in cultured human hepatocytes results in higher TG synthesis

To determine how *LPIAT1* deficiency increased cellular TG levels, cells were incubated with [^14^C]oleic acid for 24 hours, and the radioactivity in TG fractions from cells and culture medium were measured. The radioactivity in both cellular TGs, and the sum of cellular and medium TGs, corresponding to the total synthesised TGs, were increased by 30% after *LPIAT1* depletion (figure 2G). To confirm our data, we incubated HepG2 cells with [^3^H]glycerol for 5 hour, lipids were extracted and separated by one-dimensional thin layer chromatography (TLC), and radioactivity corresponding to TGs was measured (figure 2H). Consistently with our previous experiment, siRNA-mediated *LPIAT1* knockdown resulted in a 30% increase in TG synthesis (figure 2H). These results indicate that either TG synthesis or TG degradation are affected by the depletion of *LPIAT1*, without affecting TG secretion. Our results are consistent with a recent study focusing on the role of LPIAT1 on hepatic insulin resistance showing depletion of *LPIAT1* in Huh-7 results in higher TG synthesis.^23^

To assess the TG degradation, cells were incubated with [^14^C]glycerol for 12 hours, and subsequently chased with label-free medium and triacsin C to inhibit incorporation of [^14^C]glycerol into TG. TG degradation rate was determined by monitoring [^14^C]-labelled TGs at each time point. TG degradation rate was not different between *wild-type* (*WT*) and *LPIAT1* KO cells (figure 2I). Consistent with this observation, autophagy-mediated TG degradation was comparable between *WT* and *LPIAT1* KO Huh-7 cells (figure 2J). β-oxidation was also not different in *LPIAT1* KO Huh-7 cells (figure 2K) and in *LPIAT1*-depleted HepG2 cells (figure 2L). Taken all together, data suggest that *LPIAT1* downregulation/depletion results in higher intracellular TG content, specifically by increasing TG synthesis without affecting TG degradation or secretion.

Expression of most of the genes involved in TG metabolism (figure 2M) including *SREBF1* was not changed in Huh-7. Moreover, phosphorylation of Akt, which accelerates lipid synthesis (figure 2N, O), was not affected by the depletion of *LPIAT1* in this model. Finally, genes involved in TGs synthesis were not changed after siRNA-mediated *LPIAT1* knockdown in HepG2 cells (data not shown).

### Depletion of *LPIAT1* in cultured human hepatocytes results in higher PI turnover

**Figure 3:**
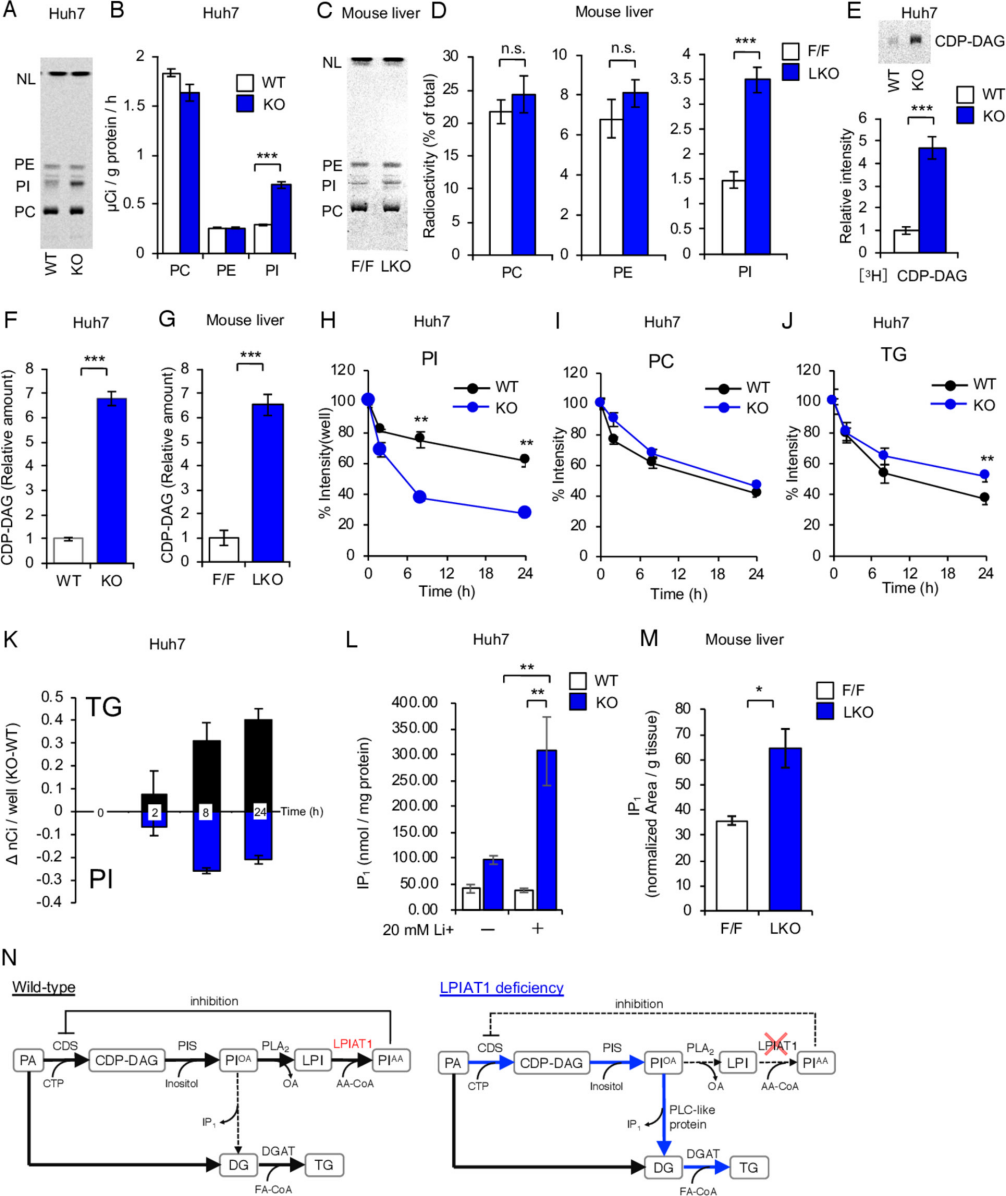
PI-derived diacylglycerol flow into TG synthesis. (A, B) Phospholipid synthesis speed measured by incorporation of [^14^C]glycerol into each phospholipid species for 2 hours. (A) Representative image of TLC separation of total lipids from WT or KO cells. (B) Radioactivity of each phospholipid fraction (n=4). (C, D) Phospholipid synthesis speed measured by incorporation of intraperitoneally injected [^14^C]glycerol (0.133 mCi/kg body weight) into liver phospholipid species of 18–20 weeks-old male F/F or LKO mice. (C) Representative image of TLC separation of total lipids from F/F or LKO mice liver. (D) Radioactivity of each phospholipid fraction (n=5). (E) CDS activity measured by the incorporation of [^3^H]CTP into CDP-DAG for 2 hours. Total lipids from WT and KO Huh-7 cells were separated by TLC and the radioactivity of CDP-DAG fraction were measured (n=4). (F) Amounts of CDP-DAG in WT and KO Huh-7 cells (n=3). (G) Amounts of CDP-DAG in F/F and LKO mice liver (n=3) (H–J) lipid degradation measured by the reduction of [^14^C]glycerol radioactivity from each lipid fraction of cells pre-incubated with [^14^C]glycerol for 12 hours and then shifted to [^14^C]glycerol-free medium at indicated time point. Radioactivity of PI (H), PC (I) and TG (J) fractions were measured. data were shown by the relative ratio of signal intensity, with the value at 0 hour set as 100% (n=3). (K) Changes in the radioactivity of PI and TG during Chase analysis. Reduction of radioactivity from 0 hour was shown at each time point (n=3). (L) Amount of cellular inositol monophosphate in WT and KO Huh-7 cells treated with or without 20 mM Li^+^ for 2 hours (n=3). (M) Amounts of inositol monophosphate in F/F and LKO mice liver intraperitoneally injected with 250 mM Li+for 3 hours (F/F, n=3; LKO, n=5) (N) A schematic diagram of the mechanism for the increase in TGs synthesis after depletion of LPIAT1. Values are shown as mean±SEM data were analysed by one-way ANOVA with Tukey’s post hoc test (L) or unpaired two-tailed Student’s t-test (B, D, E, F, G, H, I, J, M): *p<0.05, **p<0.01, ***p<0.001. ANOVA, analysis of variance; CDP-DAG, cytidine diphosphate diacylglycerol; CDS, cytidine diphosphate diacylglycerol synthase; DGAT, diacylglycerol O-acyltransferase; DG, diacylglycerol; FA, fatty acid; IP_1_, inositol monophosphate; KO, knockout; LPI, lysophosphatidylinositol; LKO, hepatocyte-specific Lpiat1 knockout; LPIAT1, lysophosphatidylinositol acyltransferase 1; NL, neutral lipid; n.s., not significant; PA, phosphatidic acid; PI^OA^, phosphatidylinositol with oleic acid; PI^AA^, phosphatidylinositol with arachidonic acid; PIS, PI synthase; PLA_2_, phospholipase A2; PLC, phospholipase C; TG, triglyceride; WT, wild-type.

To elucidate the mechanism underlying upregulated TG synthesis in *LPIAT1* KO Huh-7 cells, we analysed phospholipid metabolism. Phospholipid synthesis rate was measured by the incorporation of [^14^C]glycerol into each phospholipid fraction. The rate of [^14^C]glycerol incorporation into PI was increased by *LPIAT1* depletion, while incorporation into PC and PE did not change (figure 3A, B). PI synthesis was also upregulated in LKO mice liver (figure 3C, D). PI is produced from phosphatidic acid by sequential action of CDP-diacylglycerol (CDP-DAG) synthase (CDS) and PI synthase. mRNA levels of *CDS1*, *CDS2* and *CDIPT* (encoding PI synthase) were not significantly changed (online supplementary figure 3G). CDS2 is inhibited by AA-containing PI,^24^ and in our model the prediction would be an activation of CDS2 due to the lack of AA-containing PI. To test this hypothesis, cells were incubated with [^3^H]CTP for 2 hour, and the radioactivity in CDP-DAG fractions from cell lysates were measured. As expected, the rate of CDP-DAG synthesis was highly increased by *LPIAT1* depletion (figure 3E). Similarly, levels of CDP-DAG were strikingly increased both in *LPIAT1* KO Huh-7 cells and the liver of LKO mice, reflecting the upregulation of CDS activity (figure 3F, G, online supplementary figure 3H and I). These data suggest that depletion of AA-containing PI causes increased activity of CDS2, leading to enhanced PI synthesis in *LPIAT1* KO Huh-7 cells.

In spite of accelerated PI synthesis, cellular PI levels were comparable between *WT* and *LPIAT1* KO Huh-7 cells (online supplementary figure 3J), suggesting that *LPIAT1* depletion boosted at the same time PI synthesis and degradation. To measure PI degradation rate, we performed pulse-chase experiments with [^14^C]glycerol. After 24 hours of chase, [^14^C]-labelled PI was decreased by 73% relative to 0 hour time point in *LPIAT1 KO* cells (figure 3H). On the other hand, [^14^C]-labelled PI was decreased only by 39% in *WT* cells, indicating that *LPIAT1* depletion enhanced PI degradation rate. *LPIAT1* depletion did not affect the degradation of [^14^C]-labelled PC (figure 3I). We also analysed the degradation rate of [^14^C]-labelled TGs and found that *LPIAT1*-depletion reduced the rate of TG degradation (figure 3J). Interestingly, the difference in the amount of [^14^C]-labelled TGs between *WT* and *LPIAT1* KO cells at 24 hours of incubation matched that of [^14^C]-labelled PI at 8-hour of incubation (figure 3K). These results suggest that the glycerol moieties of degraded PI flow into TGs in *LPIAT1* KO Huh-7 cells.

PI are degraded by phospholipase C (PLC) into diacylglycerol, which is a substrate for TG synthesis. Thus, we hypothesised that PLC activity is upregulated in the *LPIAT1* KO cells and LKO liver. Inositol monophosphate (IP_1_) was accumulated in *LPIAT1* KO cells and accumulation of IP_1_ was enhanced by inhibiting IP_1_ phosphatase with Li^+^ (figure 3L). The accumulation of IP_1_ was also observed in the liver of LKO mice (figure 3M). These data suggest that PLC activity is upregulated by the absence of LPIAT1. Thus, we concluded that depletion of AA-containing PI causes activation of both PI synthesis by upregulating CDS2 activity, and PI degradation into diacylglycerol by a protein with PLC activity to generate TG (figure 3N).

### LPIAT1 depletion results in liver inflammation and fibrosis

**Figure 4:**
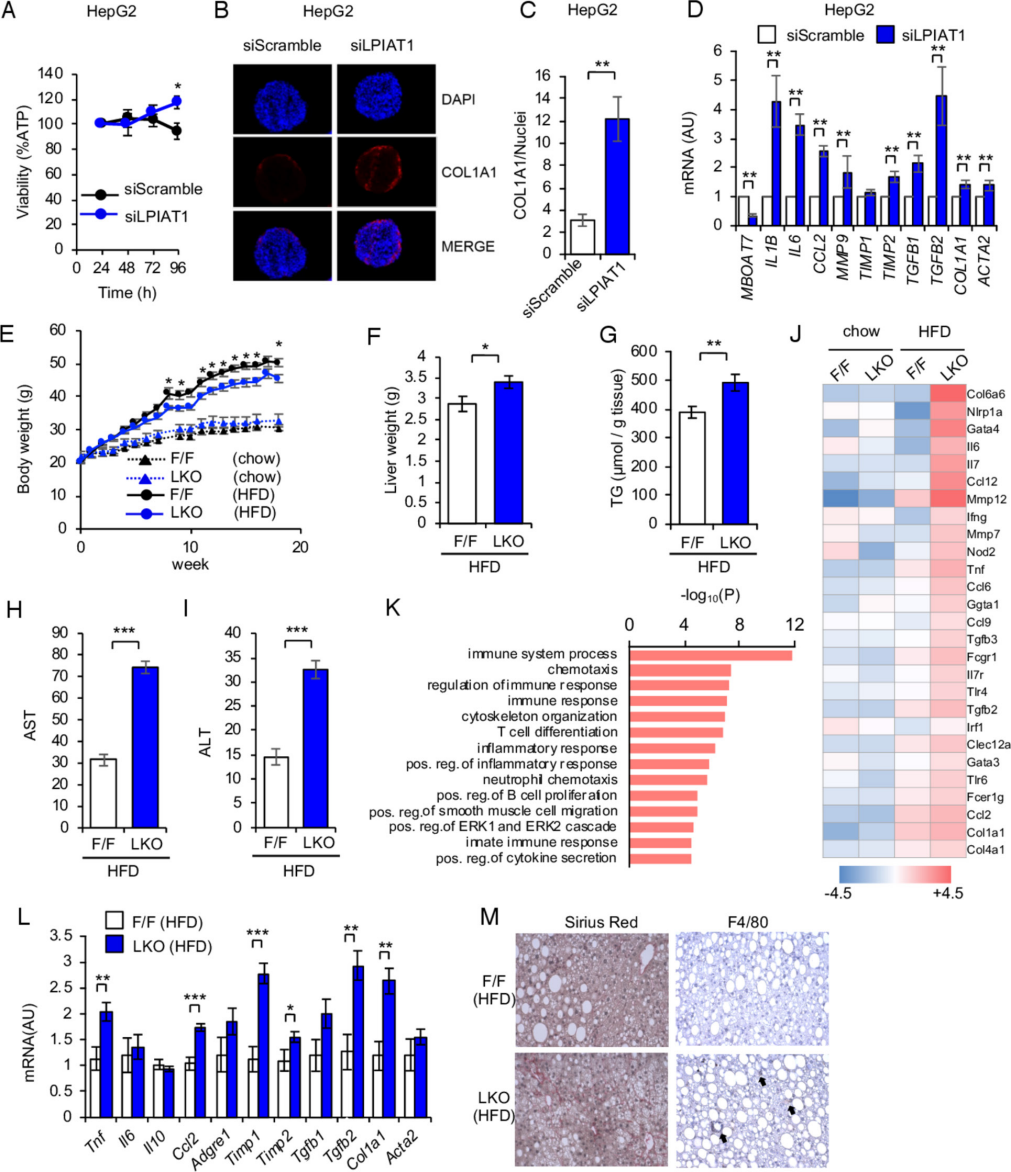
LPIAT1 depletion exacerbates HFD-driven NAFLD pathogenesis. (A–D) Spheroids were formed by coculturing HepG2 and LX-2 cells with a 24:1 ratio on ultra-low attachment plates. Cells were transiently transfected with siScramble or siLPIAT1. (A) Cellular viability normalised to the volume of spheroids (n=8). (B) Representative immunofluorescence staining of DAPI, COL1A1 and merged images of spheroids. (C) Collagen content quantified by ImageJ, normalised to number of nuclei (n=4). (D) Expression of genes involved in inflammation and fibrosis (n=5). (E–M) F/F and LKO male C57BL/6 mice were fed Chow or high fat diet (HFD) for 18 weeks. n=4 and 5 for chow-fed F/F and LKO mice. n=10 and n=13 for HFD-fed F/F and LKO mice. (E) Changes in body weights of F/F and LKO mice fed Chow or HFD (F/F, n=7–12; LKO, n=8–13). (F) Liver weights of F/F and LKO mice fed HFD (F/F, n=10; LKO, n=13). (G) Levels of hepatic TG in F/F and LKO mice (F/F, n=7; LKO, n=8). (H, I) Plasma ALT (H) and AST (I) levels of F/F and LKO mice fed HFD (F/F, n=10; LKO, n=12). (J) Selected list of differentially expressed transcripts in livers of F/F and LKO mice fed Chow or HFD. (K) Gene ontology (GO) analysis of differentially expressed transcripts in livers of F/F and LKO mice fed HFD according to the values in the enrichment score under the theme of biological processes. Top 5% of the most enriched biological processes are listed. (L) Expression of genes involved in inflammation and fibrosis in livers of F/F and LKO fed HFD. (F/F, n=6; LKO, n=9). (M) Sirius red staining and F4/80 immunostaining of liver sections from F/F and LKO mice fed HFD. Arrows indicate the sites of inflammatory cell infiltration. Images are representative of three independent experiments. Values are shown as mean±SEM data were analysed by unpaired two-tailed Student’s t-test (A, C, E, F, G, H, I, L) and non-parametric Mann-Whitney U test (D): *p<0.05, **p<0.01, ***p<0.001. DAPI, 4'6-diamidino-2-phenylindole; LKO, Lpiat1 knockout; LPIAT1, lysophosphatidylinositol acyltransferase 1; NAFLD, non-alcoholic fatty liver disease; TG, triglyceride.

*MBOAT7 rs641738 C>*T sequence variant is associated with liver fibrosis. To investigate the effect of *LPIAT1* downregulation on fibrogenesis, we downregulated *LPIAT1* by siRNA in spheroids composed by hepatocytes (HepG2 cells) and hepatic stellate (LX-2) cells.^25 26^ *LPIAT1* knockdown did not affect cell viability (figure 4A) and resulted in higher collagen synthesis by hepatic stellate cells in spheroids (figure 4B, C). We next examined mRNA expression of markers of fibrogenesis, fibrosis remodelling and inflammation. *LPIAT1* knockdown resulted in higher collagen expression, activation of stellate cells and upregulation of inflammatory and profibrotic cytokines (figure 4D). To confirm our data in vivo, *Lpiat1* ^*f/f*^ and LKO mice were fed high fat diet (HFD). LKO mice showed no differences in the relative oxygen consumption (VO_2_) (online supplementary figure 4A, B). LKO mice gained body weight similar to *Lpiat1^f/f^* mice fed chow diet during 18 weeks from 6 weeks old (figure 4E). However, when fed HFD, LKO mice gained less body weight than their *Lpiat1^f/f^* littermates. Despite the lower weight, liver weight and TG content were higher in LKO mice than in *Lpiat1^f/f^* mice (figure 4F, G). Furthermore, LKO mice showed elevated circulating levels of alanine aminotransferase (ALT) and aspartate aminotransferase (AST) when fed HFD (figure 4H, I), suggesting hepatic damage in these mice. On the other hand, there was no difference in Akt phosphorylation in the liver between *Lpiat1^f/f^* and LKO mice on insulin stimulation (online supplementary figure 4C-E).

To understand the changes associated with hepatic injury in LKO mice, hepatic gene expression from *Lpiat1^f/f^* and LKO mice fed either chow or HFD for 18 weeks were measured by microarray analysis. Consistently with our results in spheroids, categorisation of the differentially expressed genes between HFD-fed *Lpiat1^f/f^* and LKO mice, in relation to their functions in biological pathways, revealed an enrichment of genes involved in inflammation and fibrosis (figure 4J, K). Real-Time qPCR confirmed that the expression of inflammatory and fibrogenic genes were significantly higher in LKO liver than in the *Lpiat1^f/f^* liver on HFD feeding (figure 4L). Consistently, marked accumulation of extracellular collagen and inflammatory cells were observed in the section of HFD-fed LKO mice liver (figure 4M).

Taken all together, these results suggest that *LPIAT1* depletion results in NAFLD with fibrosis, recapitulating the human phenotype. Since it was proposed that phospholipid acyltransferases promote the reacylation of free AA and reduce inflammatory eicosanoid production,^6^ we investigated eicosanoid levels. Consistently with the recent work by Helsley *et al*,^23^ the levels of inflammatory bioactive lipid mediators were not affected, indicating that enhanced inflammation in LKO mice liver fed HFD were independent of inflammatory bioactive lipid mediators (online supplementary figure 4F-I).

### *LPIAT1* rs641738 variant decreases levels of *LPIAT1* mRNA and AA-containing PI in the human liver, without affecting expression of genes for de novo lipogenesis

**Figure 5:**
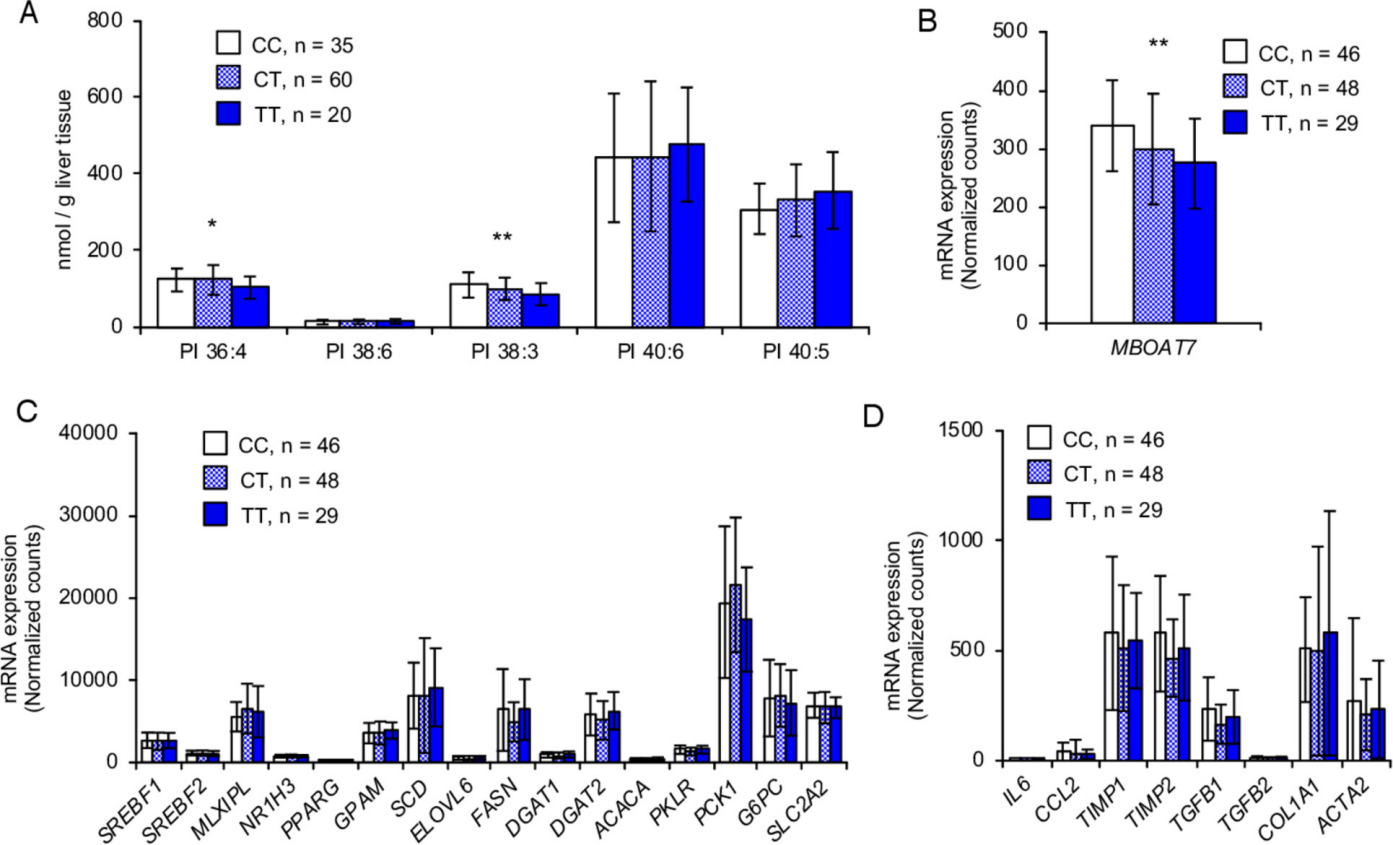
*LPIAT1* rs641738 variant decreases levels of *LPIAT1* mRNA and AA-containing PI in the human liver, without affecting expression of genes for de novo lipogenesis. (A) Lipidomic analysis of PI species in the human liver stratified by *LPIAT1* rs641738 genotype (CC, n=35; CT, n=60; TT, n=20). (B) Expression of *LPIAT1* in the human liver stratified by *MBOAT7* rs641738 genotype (CC, n=46; CT, n=48; TT, n=29). (C) Expression of genes involved in lipogenesis, β-oxidation and gluconeogenesis in human liver stratified by *LPIAT1* rs641738 genotype (CC, n=46; CT, n=48; TT, n=29). (D) Expression of genes involved in inflammation and fibrosis in human liver stratified by *LPIAT1* rs641738 genotype (CC, n=46; CT, n=48; TT, n=29). Values are shown as mean and SD. P values calculated by linear regression analysis under an additive genetic model adjusted for age, gender and BMI. Values were log transformed before entering the model. *P<0.05, **P<0.01, ***P<0.001. AA, arachidonic acid; BMI, body mass index; CC, individuals homozygote for the C allele; CT, individuals heterozygote; *LPIAT1*, lysophosphatidylinositol acyltransferase 1; *MBOAT7*, membrane bound O-acyltransferase domain containing 7; PI, phosphatidylinositol; TT individuals homozygote for the T allele.

Finally, we investigated effect of *LPIAT1* rs641738 variant on phospholipid composition and mRNA expression of genes for de novo lipogenesis and fibrosis in human liver samples. Similarly to in vitro and in vivo data, lipidomic analysis of the liver from 115 morbidly obese individuals from Finland^27^ showed that rs641738 T allele was associated with a reduction in 36:4 PI and 38:3 PI (figure 5A), probably composed by AA and mead acid in its acyl chains, respectively. On the other hand, there were no differences in other phospholipids, namely PC and PE (data not shown).

Next, we examined differences in hepatic mRNA levels of genes involved in de novo lipogenesis and fibrosis among the rs641738 genotypes in 123 morbidly obese individuals from Italy.^28^ As expected, rs641738 T allele was associated with the reduction of *LPIAT1* mRNA (figure 5B). Consistently with the results in mice, there was no difference in the liver mRNA levels of genes for de novo lipogenesis and the other pathways involved in hepatic TG handling tested among the *LPIAT1* rs641738 genotypes (figure 5C). However, there was no difference in the expression of genes related to fibrosis among the genotypes (figure 5D). This may probably due to the presence of a low degree of fibrosis in this cohort.

## Discussion

In the present study, we demonstrated that depletion of *LPIAT1* in hepatocytes causes TG accumulation fueled by an elevated PI turnover, and it promotes liver fibrosis in vivo and in in vitro liver spheroids. Although phospholipids and TGs are synthesised from a common precursor, namely phosphatidic acid, metabolic interference between phospholipids and TGs has never been shown. In this work, we show for the first time a direct metabolic flow from PI to TGs mediated by *LPIAT1* depletion in hepatocytes, causing an increase in the total hepatic TG content.

Given that PI is a minor component of membrane phospholipids (~5% of total phospholipids in the mouse liver) and that the PI amount (~63 nmol/mg protein) was far less than TG amount (~428 nmol/mg protein) in LKO liver, how does the PI breakdown match the TG accumulation in *LPIAT1*-depleted liver in terms of quantity? The metabolic labelling study in hepatocytes suggests that it is the increased PI turnover fueling quantitatively the accumulation of TGs, most probably by supplying diacylglycerol in *LPIAT1*-depleted hepatocytes.

*LPIAT1* depletion caused liver inflammation and fibrosis in mice and in human organoids without changes in inflammatory bioactive lipid mediators related to AA. A Mendelian randomisation study using common genetic variants increasing liver fat content,^29^ including the *MBOAT7 rs641738 C>T* variant, showed that increase in liver fat content causes hepatic inflammation and fibrosis. The results of the present study are consistent with this model, where excess in liver fat is per se deleterious to the liver.

NAFLD is characterised by increase in hepatic TG synthesis, mediated by SREBP1c dependent or fructose driven lipogenesis,^2^ and therapeutic targets are in development to treat NAFLD acting on this pathway.^30 31^ With our findings, we have identified a novel pathway for TG synthesis mediated by a faster PI metabolism. Future studies are warranted to understand whether the downregulation of this pathway is a viable strategy to treat NAFLD.

## Methods

### Mice

To generate *Lpiat1* floxed mice on a C57BL6/J genetic background, a~2 kb fragment of genomic DNA containing exon 2–4 of the mouse *Lpiat1* gene was flanked by two loxP sites containing the neomycin-resistant (neo^r^) cassette (mutant allele in online supplementary figure 1). The neo^r^ cassette was flanked by two FRT sites and deleted by crossing to flippase-expressing mice (floxed allele in online supplementary figure 1). Following primers were used to detect WT *Lpiat1* and *Lpiat1 floxed* alleles: P1, 5′-CACGCCCTTCACCAATGCTG-3′, P2, 5′-TGGAGGACGGTTTGCTACAGACTC-3′. Following primers were used to detect deleted alleles: P3, 5’-GGGTCATAAATGGAAGTAGAAGTA-3’, P4, 5’-TCTATAGAGTAATTTTCCTCCTTGG-3’. *Ubc-CreER^T2^* mice^19^ and *Alb-Cre* mice^20^ were obtained from The Jackson Laboratory. *Lpiat1^f/f^* mice were crossed with *Ubc-CreER^T2^* and *Alb-Cre* mice to generate *Ubc-Cre; Lpiat1^f/f^* and *Alb-Cre; Lpiat1^f/f^* (LKO), respectively. Tamoxifen was solubilised at a concentration of 20 mg/mL in corn oil (Wako) and 200 µL per 20 g body weight was injected intraperitoneally into 8-week-old male mice for 5 days to delete *Lpiat1* gene in *Ubc-Cre Lpiat1^f/f^*. All mice were housed in climate-controlled (23℃) pathogen-free facilities with a 12-hour light-dark cycle, with free access to standard chow (CE2; CLEA Japan) and water. A 6-week-old LKO and littermate control mice were placed on a HFD (HFD-60; Oriental Yeast) for 18 weeks. All animal experiments were performed in accordance with protocols approved by Animal Committees of the University of Tokyo in accordance with the Standards Relating to the Care and Management of Experimental Animals in Japan.

### Western blotting

Tissues from mice were homogenised in ice-cold lysis buffer (50 mM Tris-HCl, pH 7.4, 150 mM NaCl, 1% (v/v) TritonX-100, 0.5% (w/v) sodium deoxycholate, 0.1% (w/v) sodium dodecyl sulfate (SDS) and 20 mM ethylenediaminetetraacetic acid (EDTA)) containing protease inhibitors (10 µg/mL leupeptin, 10 µg/mL pepstatin A, 10 µg/mL aprotinin and 1 mM phenylmethylsulfonyl fluoride) and phosphatase inhibitor (5 mM NaF and 2 mM sodium orthovanadate) using Dounce homogeniser. After centrifugation at 1000 g for 20 min at 4°C, the supernatants were used as total protein extract. For Huh-7 cells, cells were washed with PBS and scraped in ice-cold PBS. After centrifugation, cell pellets were lysed in ice-cold lysis buffer and sonicated. Cells were centrifuged at 15 000 rpm for 10 min at 4°C, and the supernatants were used as total protein extract. The protein concentration was determined by the BCA assay (Pierce). Proteins were separated by SDS-polyacrylamide gel electrophoresis (PAGE) and transferred to polyvinylidene fluoride (PVDF) membranes (Millipore). After incubation with 5% (w/v) skimmed milk in TTBS buffer (10 mM Tris-HCl, pH 7.4, 150 mM NaCl, 0.05% (w/v) Tween 20) for 1 hour at room temperature, membranes were incubated with the first antibodies. The following antibodies were used: LC-3 (1/1000, MBL), p62 (1/500, Progen Bioetchnik), Akt, phospho-Akt (Ser473) and phospho-Akt (Thr308) (1/1000, Cell Signalling Technology), SREBP1(1/200, Santa Cruz Biotechnology), GAPDH (1/5000, Calbiochem). Anti-LPIAT1 was generated in our laboratory.^13^ After incubation with horseradish peroxidase-conjugated antimouse or rabbit antibody (GE Healthcare), proteins were detected by enhanced chemiluminescence (Western blotting detection system, GE Healthcare) and ImageQuant LAS 4000 system (GE Healthcare).

### Histological analysis

Tissues were fixed in Bouin’s fluid (picric acid/37% formaldehyde/acetic acid=75/25/5) or 10% neutral-buffered formalin and embedded in paraffin. The sections (5 µm thickness) were cut using a microtome and stained with H&E or stained using Picro-Sirius Red Stain kit (ScyTek Laboratories). For immunohistochemistry, the deparaffinised sections were immunostained using the antibody against F4/80 (1/50, Bio-Rad) and VECTASTAIN Elite ABC kit (Vector Laboratories) with biotinylated Rabbit Anti-Rat IgG antibody (Vector Laboratories). The sections were also counterstained with hematoxylin. For Oil Red O staining, tissues were frozen in O.C.T. Compound (Sakura Finetek) and cut using a cryostat. The sections (10 µm thickness) were allowed to dry overnight at room temperature, washed with water, rinsed with 60% isopropanol, and stained with Oil Red O solution for 15 min.

### Lipid extraction

Lipids of each tissue and cells were extracted by the method of Bligh and Dyer.^32^ The extracted solutions were dried up with centrifugal evaporator, dissolved in methanol : isopropanol=1:1, and stored at −20°C. Fatty acid metabolites were further purified from tissues by solid-phase extraction using InertSep NH2 columns (GL Science) with deuterium-labelled internal standard (11(12)-EET-d11). Briefly, InertSep NH2 columns were preconditioned with 6 mL of hexane and lipids extracted from tissues by the method of Bligh and Dyer were applied with 500 µL of chloroform. Columns were then washed with 6 mL of chloroform/isopropanol (2/1, v/v), followed by the elution with diethyl ether/acetic acid (98/2, v/v). The extracted solutions were dried up with centrifugal evaporator, dissolved in methanol:isopropanol=1:1, and stored at −20°C.

### Electrospray ionisation mass spectrometry

For the detection of phospholipids, LC/ESI-MS-based lipidomics analyses were performed on a Shimadzu Nexera UPLC system (Shimadzu) coupled with a QTRAP 4500 hybrid triple quadrupole linear ion trap mass spectrometer (AB SCIEX). Lipids extracted from tissues or cells were injected by an autosampler; typically, 10 µL (3 nmol phosphorous equivalent) of the sample was applied. Chromatographic separation was performed on a SeQuant ZIC-HILIC PEEK coated column (250 mm × 2.1 mm, 1.8 µm; Millipore) maintained at 50°C using mobile phase A (water/acetonitrile (95/5, v/v) containing 10 mM ammonium acetate) and mobile phase B (water/acetonitrile (50/50, v/v) containing 20 mM ammonium acetate) in a gradient programme (0–22 min: 0% B→40% B; 22–25 min: 40% B→40% B; 25–30 min: 0% B) with a flow rate of 0.5 mL/min. The instrument parameters for negative ion mode were as follows: curtain gas, 10 psi; collision gas, 7 arb. unit; ionspray voltage, −4500 V; temperature, 700°C; ion source gas 1, 30 psi; ion source gas 2, 70 psi. The instrument parameters for positive ion mode were as follows: curtain gas, 10 psi; collision gas, 7 arb. unit; ionspray voltage, 4500 V; temperature, 700°C; ion source gas 1, 30 psi; ion source gas 2, 50 psi. Precursor ion scan of 184 Da with a scan range of m/z 510–960 for Q1 in the positive ion mode were used to detect PC and SM. Neutral loss scans of 141 Da with a scan range of m/z 510–960 for Q1 in the positive ion mode were used to detect PE. Precursor ion scan of m/z 241 and 153, and neutral loss scans of 87 Da with a scan range of m/z 550–1000 for Q1 in the negative ion mode was used to detect PI (LPI), PG and PS, respectively. Detection of CDP-DAG species was performed by multiple reaction monitoring (MRM) as described in online supplementary table 1.

For the detection of fatty acid metabolites, chromatographic separation was performed on a ACQUITY UPLC HSS T3 column (2.1×100 mm, 1.8 µm; Waters) maintained at 40°C using mobile phase A (water/acetic acid (100/0.1, v/v) containing 10 mM ammonium acetate) and mobile phase B (acetonitrile/methanol (4/1, v/v) containing 10 mM ammonium acetate) in a gradient programme (0–2 min: 90% A; 2–10 min: 90% A →30% A; 10–24 min: 30% A →27% A; 24–27 min: 1% A; 27–32 min: 90% A) with a flow rate of 0.2 mL/min(0–10 min), 0.1 mL/min(10–15 min),0.2 mL/min(15–24 min) and 0.5 mL/min(24–32 min). The instrument parameters were as follows: curtain gas, 10 psi; ionspray voltage, −4500 V; temperature, 600°C; ion source gas 1, 70 psi; ion source gas 2, 80 psi. The specific detection was performed by MRM as described in online supplementary table 1.

IP_1_ was detected as previously reported.^33^ Chromatographic separation was performed on a Biobasic AX column (2.1 × 150 mm, 5 µm; Thermo) maintained at 25°C using mobile phase A (water/methanol (95/5, v/v)) and mobile phase B (200 mM ammonium carbonate; pH 9.0) in a gradient programme (0–4 min: 100% A; 4–6 min: 100% A → 80% A; 6–20 min: 80% A → 45% A; 20–45 min: 100% A) with a flow rate of 0.2 mL/min. The instrument parameters were as follows: curtain gas, 10 psi; collision gas, 7 arb. unit; ionspray voltage, −4500 V; temperature, 700°C; ion source gas 1, 30 psi; ion source gas 2, 70 psi. IP_1_ detection was performed by MRM using the transition m/z 259>97 in negative mode.

### Determination of phospholipid composition

Phospholipids were separated by TLC on silica gel 60F254 plates (Merck) in chloroform:methanol:acetic acid=65:25:13. The area of silica gel corresponding to each phospholipid (SM, PC, PS+PI, PE, PG and CL) was scraped off the plates. Isolated phospholipids were then extracted by the method of Bligh and Dyer described above. The fraction containing PS and PI was further separated by TLC in chloroform:methanol:formic acid:water=60:30:7:3, then PS and PI were extracted separately. To determine the amount of each phospholipid, lipid phosphorus was measured by Bartlett’s method.^34^

### Measurement of cellular or hepatic TG and cholesterol level

TG E Test Wako and Cholesterol E Test Wako (Wako) were used respectively according to manufacturer’s instruction. In brief, lipid extracts from tissues or cells were dried in a glass and added 15% TritonX-100 in acetone followed by drying up and incubated with reaction solution for 5 min. Absorption at 600 nm was measured and TG and cholesterol levels were calculated.

### Real-time PCR

Total RNA was extracted from tissues and cells using Isogen II (Nippon-gene) and reverse-transcribed using the high-capacity complementary DNA (cDNA) reverse-transcriptase Kit (Applied Biosystems). Quantitative real-time PCR was carried out using SYBR Green PCR Master Mix or KAPA CYBR FAST qPCR Master mix (TaKaRa) and LightCycler 480 or LightCycler 96 (Roche Diagnostics). All measurements were normalised to GAPDH, β-actin or 18S ribosomal RNA.

For gene expression analysis of spheroids, cells were subjected to RNA extraction by RNeasy Plus kit (Qiagen, Hilden, Germany). RNA was retrotranscribed by high-capacity cDNA reverse transcription kit (ThermoFisher Scientific). Gene expression was detected by real-time qPCR (Bio-Rad Laboratories), using TaqMan probes (Life Technologies).

The probe-primer sets and the sequences of the oligonucleotides are listed in online supplementary tables 2 and 3.

### Measurement of hepatic TG secretion in vivo

Measurement of hepatic TG secretion rate was performed as previously reported^21^ with slight modification. Mice were fasted from AM 10:00 for 6 hours and intraperitoneally injected with 200 µL of 1 mg/mL Poloxamer 407 (P-407) (Sigma) solution in sterile PBS. Blood was collected prior to injection (0 hour) and at 1.5 and 3 hours after injection. Serum TG was measured using TG E Test Wako.

### PCR cloning

Mouse *Lpiat1* were amplified by PCR using cDNA derived from brain of C57BL/6J mice as templates.

### Measurement of plasma biomedical markers and lipoprotein profiles

Blood samples were collected with EDTA from the mice fasted for 6 hour. Plasma lipoprotein profiles were analysed by an online dual enzymatic method using high-performance liquid chromatography (Skylight Biotech). Serum ALT and AST levels were quantified using the transaminase CII-test Wako kit (Wako). Serum TG or cholesterol levels were quantified using the TG E Test Wako and Cholesterol E Test Wako (Wako), respectively. Serum glucose level was quantified using Glucose CII-test Wako kit (Wako). Serum insulin level was quantified using mouse insulin ELISA kit (Mercodia).

### Microarray

Total RNA was extracted from LKO or F/F mice liver and purified using the RNeasy Mini Kit (Qiagen). Microarray was performed by SurePrint G3 Mouse gene expression 8×60K Ver. 2.0 (Agilent Technologies).

### Oxygen consumption

VO_2_ was measured with an O_2_/CO_2_ metabolism measurement system every 3 min for 24 hours in the animals under the fasting condition (Model MK-5000; Muromachikikai). Each mouse was placed in a sealed chamber (560 mL volume) with an air flow of 0.5 L/min for 24 hours at room temperature. The oxygen consumed was converted to millilitres per minute by multiplying it by the flow rate.

### Insulin sensitivity

HFD-fed mice were fasted for 6 hour and injected with or without insulin (Eli Lilly) (5 mU/g body weight) for 10 min. Liver were homogenised and Akt phosphorylations were analysed by western blot.

### Cell culture

Huh-7 cells and HepG2 were obtained from JCRB cell bank and ATCC, respectively. LX-2 cells were purchased form Millipore. Huh-7 cells and LX-2 cells were maintained in Dulbecco’s modified Eagle’s medium (DMEM) supplemented with 10% foetal calf serum and 100 units/mL penicillin 100 µg/mL streptomycin, and 2 mM L-glutamine (PSG). HepG2 cells were maintained in minimum essential medium (MEM) supplemented with 10% FBS, 2 mM L-glutamine, 1 mM sodium pyruvate, 1x non-essential amino acids, 100 units/mL penicillin and 100 µg/mL streptomycin. These cell lines have been tested for mycoplasma contamination.

### Spheroids culture

HepG2 and LX-2 cells were cocultured into 96-well round bottom ultralow attachment plates (Corning,) at 2000 viable cells per well, with a 24:1 ratio. Cells were transiently transfected with 5 nM Scramble siRNA (#AM4635; Thermo Fisher) or 5 nM *LPIAT1* siRNA (S35614, S35615, S35616; Thermo Fisher). Cells were grown in MEM +10% FBS for 96 hours at 37°C in a humidified atmosphere of 5% CO_2_. Cellular ATP levels were analysed using the CellTiter-Glo Luminescent Cell Viability Assay (Promega) after 24, 48, 72 and 96 hours. ATP levels were normalised to spheroids volumes. Spheroid pictures were taken by Axio Vert.A1 inverted microscope (Carl Zeiss AG).

### Generating LPIAT1 KO Huh-7 cells

Single guide RNA (sgRNA) sequences targeting the region adjacent to the exon 3 of *LPIAT1* were designed using the online CRISPR design tool from the Zhang lab at the Broad Institute. The sgRNA sequence, listed in online supplementary table 4, was then cloned into the pSpCas9 (BB)−2A-Puro (PX459) vector (a gift from Feng Zhang, Addgene plasmid # 48139).^35^ These vectors were transfected to Huh-7 cells using Lipofectamine 2000 (Invitrogen). Forty-eight hours after transfection, the cells were selected with fresh medium containing 1 µg/mL puromycin.

### Retrovirus-mediated gene expression and selection

For retroviral gene expression, GFP-tagged LPIAT1 was introduced into a pMXs-Puro Retroviral Vector (Cell Biolabs). These retroviral vectors and VSVG encoding vector were transfected to a transient retrovirus packaging cell line PlatE (provided by T. Kitamura, Institute of Medical Science, University of Tokyo) using Lipofectamine 2000 (Invitrogen). Forty-eight hours after transfection, retrovirus was collected by centrifugation. For infection into Huh-7 cells, the cells were incubated with the retrovirus for 24 hours in the presence of 10 µg/mL polybrene (Sigma-Aldrich). Infected Huh-7 cells were selected with fresh medium containing 1.5 µg/mL puromycin.

### BODIPY staining

Cells grown on coverslips were fixed with 4% paraformaldehyde in PBS at room temperature for 15 min, permealised with 0.1% Triton X-100 in PBS at room temperature for 5 min. Lipid droplets were stained with BODYPY 493/503 (1/100 in 3% BSA in PBS) (Invitrogen) for 1 hour. Confocal microscopy was performed using a TCS SP8 (Leica) with a 63 × 1.2 Plan-Apochromat oil immersion lens.

### Oil red O staining

HepG2 cells were transiently transfected with Scramble siRNA 5 nM or *LPIAT1* siRNA 5 nM. Cells were grown in MEM +10% FBS at 37°C in a humidified atmosphere of 5% CO_2_. After 48 hours, HepG2 cells were harvested and subjected to Oil Red O fluorescent staining (Sigma-Aldrich). Nuclei were staining using DAPI (Sigma-Aldrich). Neutral lipid content was normalised to the number of nuclei, and quantified by ImageJ.

### Collagen content determination by immunofluorescence

Spheroids were cultured as described above, and collected after 96 hours. Spheroids were subjected to immunofluorescence for the determination of the collagen content, as previously described.^26^ Briefly, Sections were blocked with 4% BSA-PBS for 1 hour and then stained with anti-COL1A1 (Sigma-Aldrich, HPA011795) (1/100) in 4% BSA-PBS for 1 hour, followed by incubation with fluorescent secondary antibody (anti-rabbit IgG Alexa Fluor 594, invitrogen) (1/1000) for 1 hour at room temperature. Nuclei were stained by DAPI (Sigma-Aldrich) (1/8000 in PBS) for 5 min. Pictures were obtained using Axioplan 2 (Zeiss) with AxioVision 4.8 Software (Zeiss). Collagen content was normalised to the number of nuclei, and quantified by ImageJ.

### Analysis of TG synthesis

Huh-7 cells were incubated in DMEM supplemented with 10% foetal calf serum and PSG containing 0.4 µCi per well of [^14^C]oleic acid for 24 hours, followed by lipid extraction and separation by TLC in hexane/diethyl ether/acetic acid (70/30/1, v/v). Radioactivity of TG fraction was measured.

HepG2 cells were incubated with MEM w/o FBS +6 µCi/mL [^3^H]glycerol (PerkinElmer) +1.5 mM glycerol (Sigma-Aldrich)+1% BSA (Sigma-Aldrich) for 5 hour. Cells were collected and washed with PBS to remove the excess of medium. Lipids were extracted adding 3 mL of chloroform:methanol (2:1 v/v) (Sigma-Aldrich) and 1 mL of acidified solution (17 mM NaCl, 1 mM H_2_SO_4_) (Sigma-Aldrich). Samples were centrifuged at 3000 rpm for 10 min. The lower organic phase was saved and dried under a gentle nitrogen stream. Lipids were reconstituted in 50 µL of chloroform (Sigma-Aldrich), and separated by one-dimensional TLC using TLC silica gel plates (Merck-Millipore). Triolein (Sigma-Aldrich) was used as a marker. Petroleum ether:diethyl ether:acetic acid (40:60:1, vol/vol) (Sigma-Aldrich) was used as the mobile phase. The area of TLC silica gel corresponding to the newly synthesised radio-labelled TG were cut out. The rate of released radio-labelled TG was measured, using liquid scintillation counting (PerkinElmer). Data were normalised for number of cells.

### Analysis of phospholipid synthesis

Huh-7 cells were incubated in DMEM supplemented with 10% foetal calf serum and PSG containing 0.4 µCi per well of [^14^C]glycerol for 2 hours. For analysis of CDP-DAG synthesis, cells were incubated in inositol depleted DMEM with 2% dialyzed FBS and PSG containing 10 µCi [^3^H]CTP for 2 hours. Cell lipids were extracted and separated by TLC in chloroform/1-propanol/ethyl acetate/methanol/0.25% KCl (25/25/25/10/9, v/v) and radioactivity of each phospholipid fraction was measured.

### Analysis of phospholipid synthesis in vivo

Eighteen-to-twenty-week-old mice were fasted for 6 hours before intraperitoneal injection of [^14^C]glycerol for 30 min (0.133 mCi/kg body weight). Mice were killed by cervical dislocation and liver was immediately frozen. Radioactivity of each phospholipid fraction was measured as mentioned above.

### Analysis of lipid degradation speed

Huh-7 cells were preincubated in DMEM supplemented with 10% foetal calf serum and PSG containing 0.4 µCi per well of [^14^C]glycerol for 12 hours, and then shifted to the [^14^C]glycerol-free medium. After 2, 8, 24 hours of incubation with [^14^C]glycerol-free medium, cell lipids were extracted and separated by TLC in chloroform/1-propanol/ethyl acetate/methanol/0.25% KCl (25/25/25/10/9, v/v) for phospholipid and hexane/diethyl ether/acetic acid (70/30/1, v/v) for TG and radioactivity of each phospholipid fraction were measured.

### β-oxidation assay

β-oxidation ability was determined as described previously.^36^ After incubation of Huh-7 cells with 1 µCi [^14^C]palmitic acid for 4 hour, cells were harvested and the cell pellets were suspended with 1 M perchoric acid, vortex-mixed for 5 min and centrifuged at 14 000 g for 10 min at 4°C. Radioactivity of the water soluble fraction was quantified by liquid scintillation counting.

HepG2 cells were incubated with MEM w/o FBS +8.3 µCi/mL [^3^H]palmitate (PerkinElmer) +2.2 mM PA (Avanti Polar Lipids)+1% BSA for 2 hours. Cell media were collected and palmitate β-oxidation rate was assessed, as previously described by Hansson *et al*.^37^ The rate of oxidised [^3^H]palmitate was measured using liquid scintillation counting (PerkinElmer). Data were normalised for number of cells.

### Fatty acid composition of TG

TG were separated from lipid extracts of Huh-7 cells by TLC in hexane/diethyl ether/acetic acid (70/30/1, v/v). The area corresponding to TG was scraped off the plates and TG was extracted from silica gel. Isolated TG were methylated with 2.5% H_2_SO_4_ in methanol. The resulting fatty acid methyl esters were then extracted with hexane and subjected to GC-MS analysis as described previously.^38^ GC-MS analysis was performed by using an Agilent 7890A-5975C GC-MS network system (Agilent Technologies, Wilmington, Delaware, USA) equipped with a DB-23 capillary column (60 m × 250 μm × 0.15 μm; Agilent Technologies). The oven temperature programme was as follows; the initial temperature was 50°C for 1 min, then raised to 175°C at 25°C/min, to 235°C at 5 °C/min, and held for 5 min. The injector and detector temperatures were both set at 250°C.

### Extraction of inositol phosphate

IP_1_ extraction was performed as previously reported^39 40^ with brief modification. Cell pellets were suspended with 3.2 M acetic acid in methanol and vortex-mixed for 30 min at room temperature and centrifuged at 5000 g for 20 min at 4°C. Supernatant was dried up with centrifugal evaporator and resuspended in 100 µL 5% aqueous methanol. 10 µL were used to determine the content of inositol phosphate. Mice liver were homogenised in extraction buffer (1 M HClO_4_, 3 mM EDTA) using handy micro homogeniser (Physcotron NS-310E). After centrifugation at 15 000 rpm for 5 min at 4°C, the supernatants neutralised with neutralisation buffer (1 M K_2_CO_3_, 3 mM EDTA) and incubated on ice for 2 hours. After centrifugation at 15 000 rpm for 10 min at 4°C, supernatants were collected and 5 µL were used to determine the content of inositol phosphate.

### Autophagy flux assay

For induction of autophagy, the culture medium was changed to glucose-free and amino acid-free EBSS for 4 hours with or without 150 nM Bafilomycin A1 (Sigma). LC3 protein was detected by western blotting.

### Lipidomic and gene expression analysis in the human liver

The cohort from Finland where the genetic association between the *MBOAT7* rs641738 and lipidomic data were examined has been previously described.^27^ Lipidomic data were obtained from 125 human liver biopsy using a quadrupole time-of-flight (Q-TOF) Premier mass spectrometer combined with an Acquity Ultra Performance LC (Waters, Milford, Massachusetts, USA) as previously described.^27^ Briefly, immediately at the beginning of the laparoscopic bariatric surgery, wedge biopsies of the liver were obtained from obese adults. Part of the biopsy was snap-frozen in liquid nitrogen for subsequent analysis of molecular lipids. *MBOAT7* rs641738 genotype was performed by TaqMan PCR method (Applied Biosystems, Foster City, California, USA) according to the manufacturer’s instructions followed by post-PCR allele-specific fluorescence measurements by an ABI Prism Sequence Detection System (ABI 7900HT, Applied Biosystems). In the present study, only results on liver phospholipids composition from a subset of 115/125 individuals with complete required data were included. Each participant provided written informed consent after being explained the nature and potential risks of the study.

The cohort from Italy where the genetic association between the *MBOAT7* rs641738 and liver mRNA expression was examined has been previously described.^28^ Gene expression data were obtained from human liver biopsy of a subset of adult obese individuals as previously described.^25 41^ Briefly, RNA sequencing was performed using the Illumina HiSeq 4000 platform (Novogene, Hong Kong, China). RNA reads were mapped against the human genome, and the gene read count (Ensembl human transcript reference assembly, V.75) was determined using RSEM software. To quantify gene expression, the RSEM per gene count data were normalised using the DESeq2 package. *MBOAT7* rs641738 genotype was performed by TaqMan PCR method (Applied Biosystems, Foster City, California, USA) as previously described.^8^

### Statistical analysis

For in vivo and in vitro studies, data were converted to means±SEM., and the unpaired Student’s t-test or non-parametric Mann-Whitney test were applied to determine significant differences between two samples. Statistical differences between multiple treatment groups and a control group were determined using analysis of variance and the Tukey-Kramer post hoc test. Sample sizes were chosen on the basis of previous experience in our laboratory. The experiments were performed and analysed in a non-randomised and non-blinded fashion. No data were excluded from the analysis. Variance was similar between the groups that were statistically compared.

Data from lipidomic composition and gene expression in human liver tissues stratified by *MBOAT7* rs641738 genotype were shown as mean and SD. P values were calculated by linear regression analysis under an additive genetic model adjusted for age, gender and body mass index. Values were log transformed before entering the model. Statistical analyses were performed using IBM Statistical Package for Social Sciences (IBM SPSS, V.19.0).

### Data availability

The data supporting the findings of this study are available from the corresponding author on reasonable request. The microarray data can be accessed at the Gene Expression Omnibus repository (GSE141920). Primer sets for real-time PCR, probes for qPCR by the TaqMan system and sgRNA target sequences are available in online supplementary tables 2-4, respectively.
