# Supplementary material for: LPIAT1/MBOAT7 depletion increases triglyceride synthesis fueled by high phosphatidylinositol turnover
Source: Gut. 2020 Apr 6;70(1):180–93. doi: 10.1136/gutjnl-2020-320646 (PMC7788230; doi:10.1136/gutjnl-2020-320646)
Supplement: Supplementary data [file gutjnl-2020-320646supp001.pdf]

Supplementary Figure S1

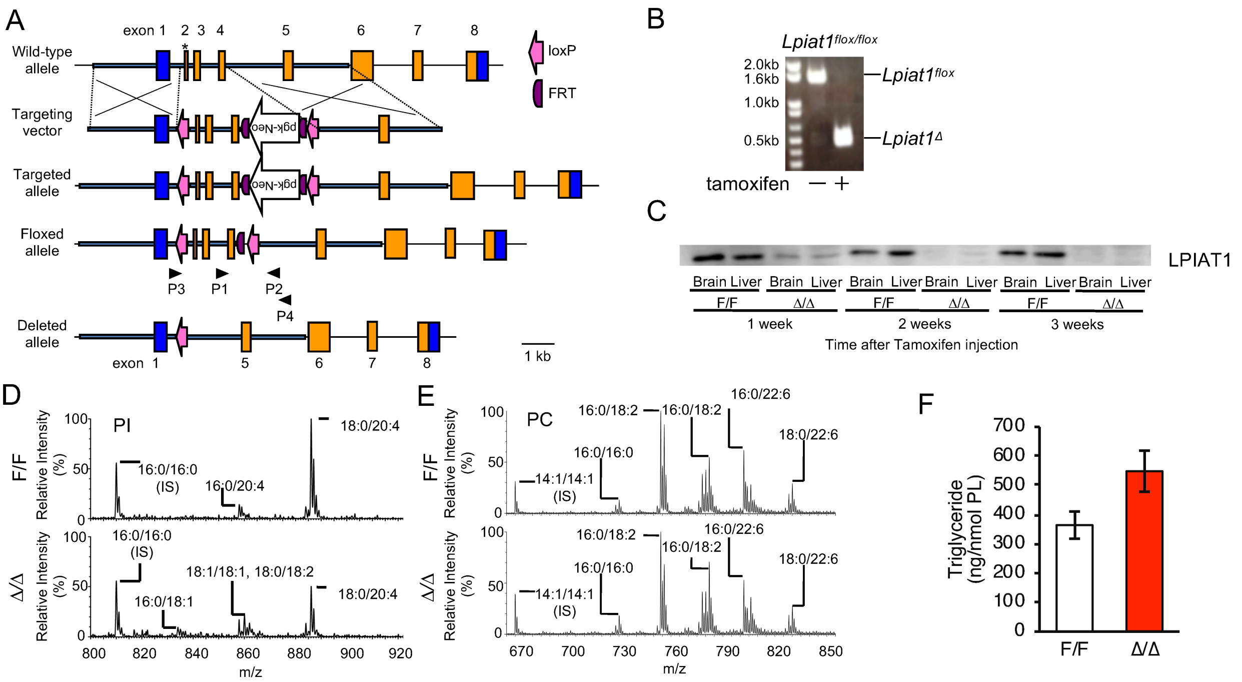

Supplementary Figure S1.
Generation of tamoxifen-inducible *Lpiat1* knockout mice.
(*A*) Diagram of the *Lpiat1* genomic locus and the targeting vector. The positions of PCR primers are indicated (P1, P2, P3 and P4) under the diagram of Floxed allele. The mice carrying Floxed allele and deleted allele homozygously in whole bodies are shown as F/F and Δ/Δ respectively. Liver-specific *Lpiat1* deleted mice are shown as LKO. (*B*) PCR analysis of genomic DNAs from F/F mice injected with tamoxifen or vehicle (corn oil). Similar results were obtained in one additional independent experiment. (*C-F*) Six weeks old male *Lpiat1$^{f/f}$* mice (F/F) and *Ubc-CreER^{T2};Lpiat1$^{f/f}$* mice (Δ/Δ) were treated with tamoxifen for an indicated weeks (*C*) or 4 weeks (*D-F*). (*C*) Western blot of LPIAT1 in brain and liver from F/F and Δ/Δ mice treated with tamoxifen. Similar results were obtained in one additional independent experiment. (*D,E*) LC-MS spectra of PI (*D*) and PC (*E*) species of F/F (top) and Δ/Δ (bottom) liver. Data are representative of three independent experiments. (*F*) Hepatic triglyceride levels of F/F and Δ/Δ mice treated with tamoxifen (n=3). Values are shown as mean ± SEM.

Supplementary Figure S2

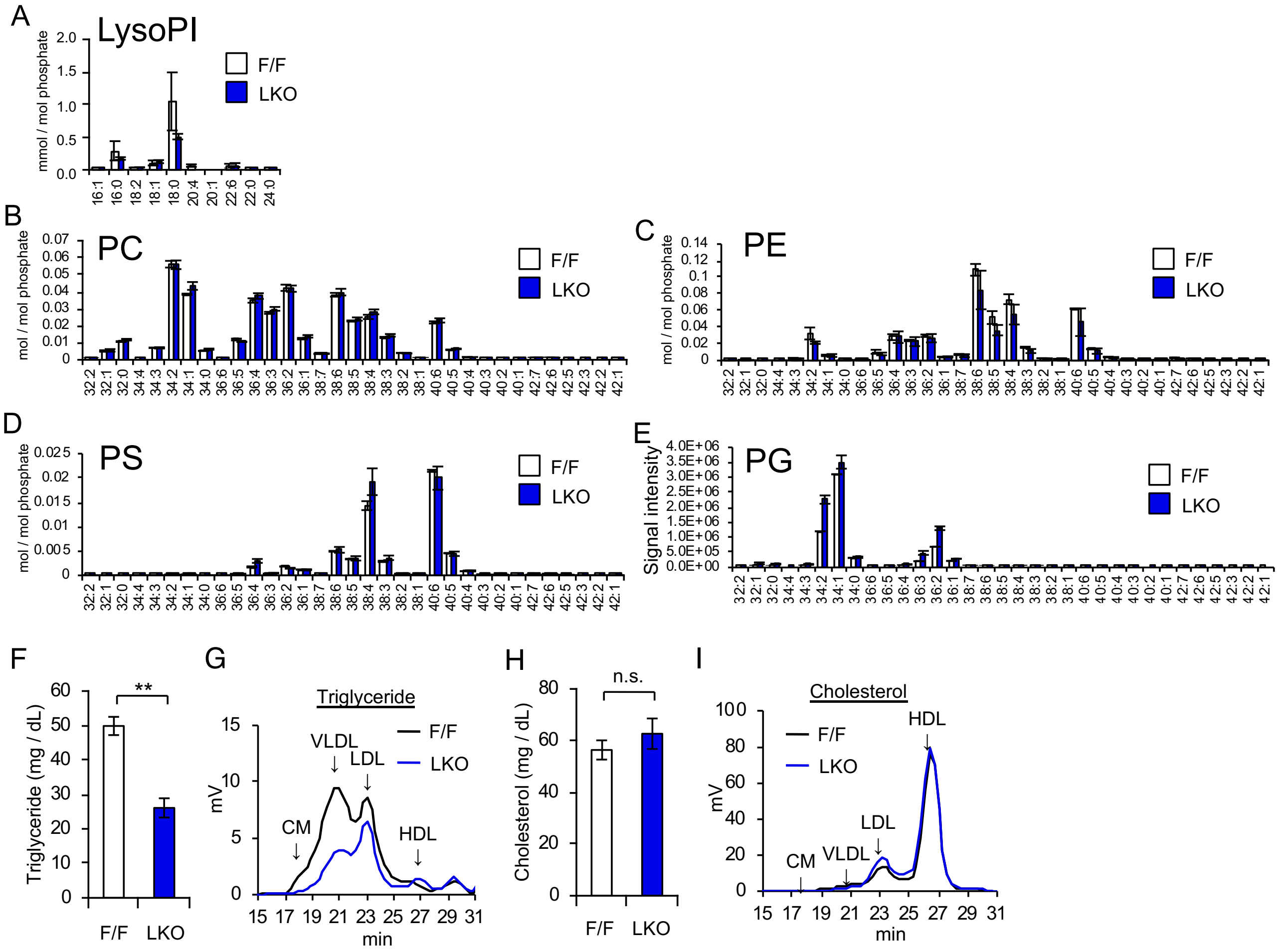

Supplementary Figure S2.
Lipid profiles of hepatocyte-specific *Lpiat1* knockout mice.
(*A-E*) LC-MS/MS analysis of lyso-phospholipid and phospholipid species in the livers of F/F and LKO mice (n=3). (*F-I*) Plasma triglyceride levels (*F, G*) and cholesterol levels (*H, I*) of F/F and LKO mice (n=3). Lipoprotein profiles of plasma from 12-week-old male F/F and LKO mice after 6 h of starvation analyzed by HPLC. CM; chylomicron, VLDL; very low-density lipoprotein, LDL; low-density lipoprotein, HDL; high-density lipoprotein. Values are shown as mean ± SEM. Data were analyzed by unpaired two-tailed Student's *t* test (*A, B, C, D, E, F, H*): *p<0.05 and **p<0.01. n.s., not significant.

Supplementary Figure S3

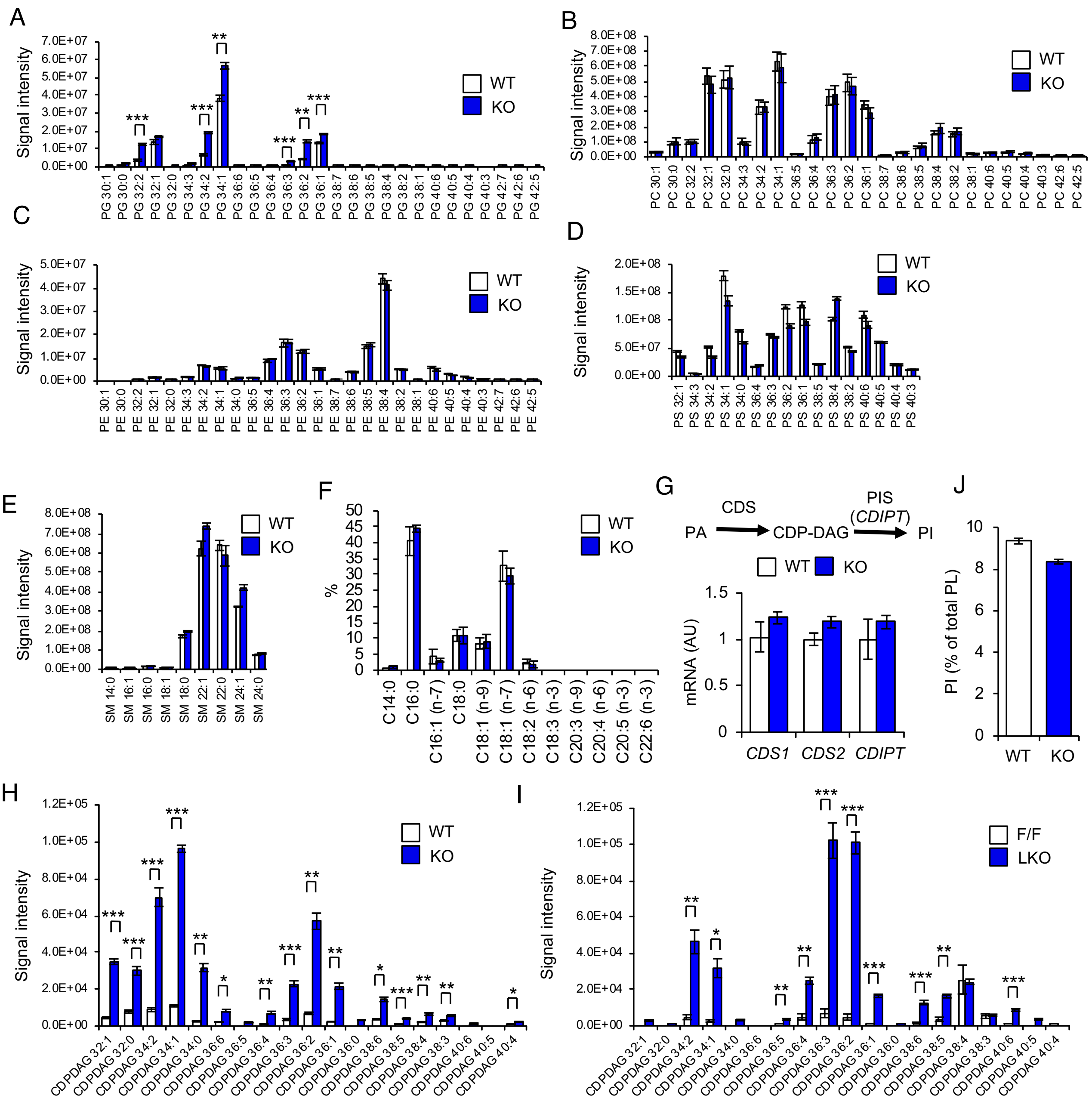

Supplementary Figure S3.
Lipid profiles of *LPIAT1* knockout Huh-7 cells.
(*A-E*) LC-MS/MS analysis of PG (*A*), PC (*B*), PE (*C*), PS (*D*), and SM (*E*) species in WT and KO Huh-7 cells (n = 3). (*F*) Fatty acid esterified to cellular TG of WT and KO cells. Data are expressed as mol% of total fatty acids esterified to TG (n = 3). (*G*) A scheme diagram of PI synthesis and expression of genes involved in PI synthesis in WT and KO Huh-7 cells (n = 3). (*H,I*) LC-MS/MS analysis of CDP-DAG species in WT and KO Huh-7 cells (*H*) and in F/F and LKO mice liver (*I*) (n = 3). (*J*) Amount of PI from WT and KO Huh-7 cells (n = 3). Values are shown as mean ± SEM. Data were analyzed by unpaired two-tailed Student's *t* test (*A, B, C, D, E, F, G, H, I, J*): $^{**}p$<0.01 and $^{***}p$<0.001.

Supplementary Figure S4

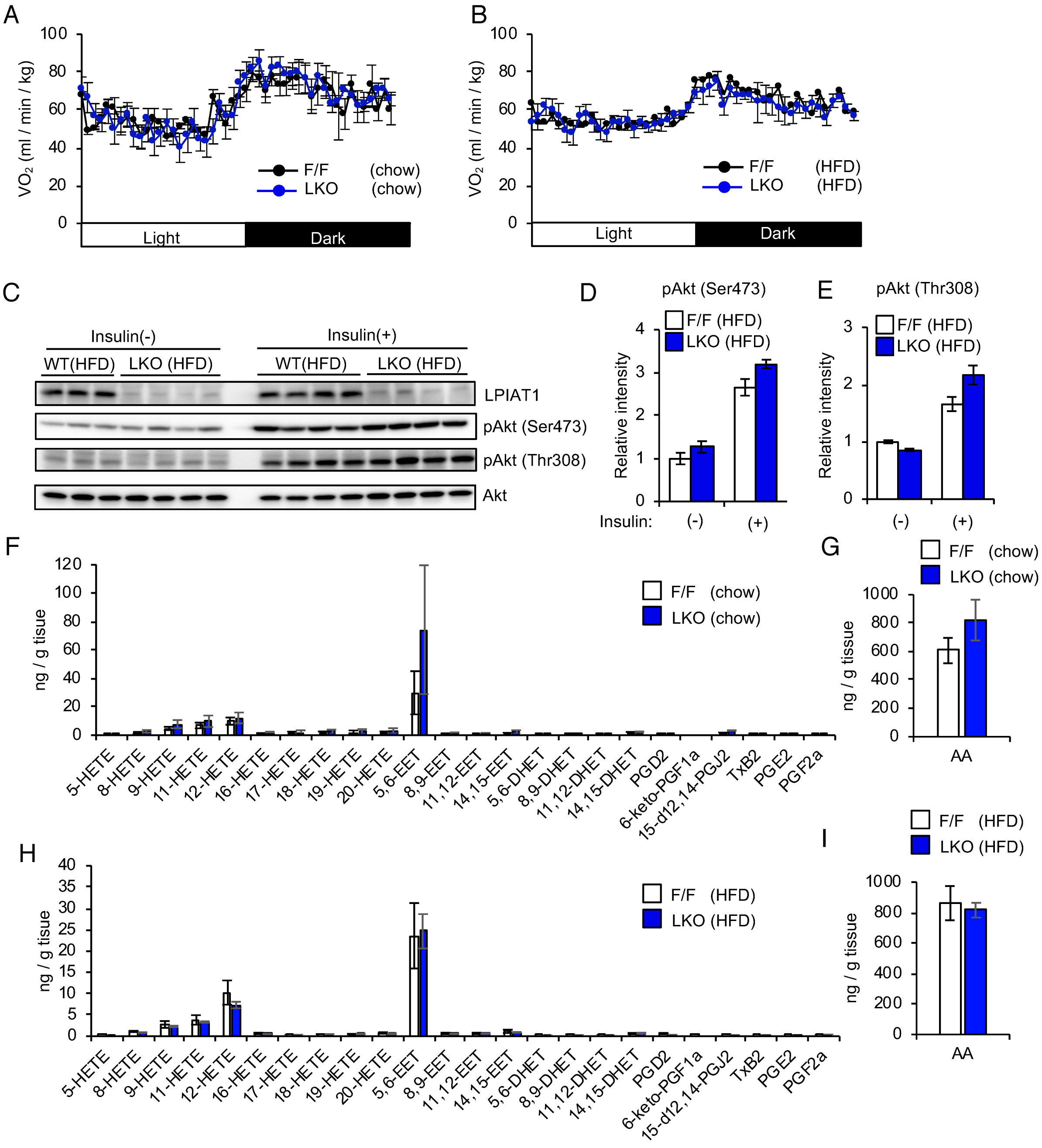

Supplementary Figure S4.

Oxygen consumption and liver eicosanoid levels in hepatocyte-specific *Lpiat1* knockout mice fed chow or high fat diet. (*A,B*) VO_2 of F/F and LKO mice fed chow (*A*) or HFD (*B*) (n = 5). 8-week-old mice and 12-week-old mice (HFD feeding from 8-week-old) were used to measure VO_2. (*C*) Western blot analysis of Akt phosphorylation in F/F and LKO mice fed HFD with or without intraperitoneal injection of insulin (5 mU / g body weight) for 10 min. (*D,E*) The ratios of p-Akt (Ser473) (D) and p-Akt (Thr308) (E) to Akt were measured, with the value of WT being as 1 (n = 3-4). (*F,G*) Quantification of arachidonic acid (AA) derived mediators (*F*) and AA (*G*) of chow-fed F/F and LKO mice liver (n = 4). (*H,I*) Quantification of AA-derived mediators (*H*) and AA (*I*) of HFD-fed F/F and LKO mice liver (F/F, n = 3; LKO, n = 5). Values are shown as mean ± SEM. Data were analyzed by unpaired two-tailed Student's *t* test (*A, B, D, E, F, G, H, I*).

Supplementary Table S1. Optimized MRM pairs and parameters for lipids.

	m/z		DP (V)	EP (V)	CE (V)	CXP (V)
	Precursor	Product				
CDPDAG 32:1	950.5	384	-180	-10	-58	-11
CDPDAG 32:0	952.5	384	-180	-10	-58	-11
CDPDAG 34:2	976.5	384	-180	-10	-58	-11
CDPDAG 34:1	978.5	384	-180	-10	-58	-11
CDPDAG 34:0	980.5	384	-180	-10	-58	-11
CDPDAG 36:6	996.5	384	-180	-10	-58	-11
CDPDAG 36:5	998.5	384	-180	-10	-58	-11
CDPDAG 36:4	1000.5	384	-180	-10	-58	-11
CDPDAG 36:3	1002.5	384	-180	-10	-58	-11
CDPDAG 36:2	1004.5	384	-180	-10	-58	-11
CDPDAG 36:1	1006.5	384	-180	-10	-58	-11
CDPDAG 36:0	1008.5	384	-180	-10	-58	-11
CDPDAG 38:6	1024.5	384	-180	-10	-58	-11
CDPDAG 38:5	1026.5	384	-180	-10	-58	-11
CDPDAG 38:4	1028.5	384	-180	-10	-58	-11
CDPDAG 38:3	1030.5	384	-180	-10	-58	-11
CDPDAG 40:6	1052.5	384	-180	-10	-58	-11
CDPDAG 40:5	1054.5	384	-180	-10	-58	-11
CDPDAG 40:4	1056.5	384	-180	-10	-58	-11

	m/z		DP (V)	EP (V)	CE (V)	CXP (V)	Retention time (min)
	Precursor	Product					
AA	303	259	-110	-10	-18	-9	25.95
5-HETE	319	115	-45	-10	-22	-21	19.68
8-HETE	319	155	-50	-10	-18	-17	19.35
9-HETE	319	123	-35	-10	-24	-11	19.49
11-HETE	319	167	-50	-10	-22	-13	19.11
12-HETE	319	179	-60	-10	-20	-7	19.38
16-HETE	319	233	-55	-10	-20	-9	18.29
17-HETE	319	247	-55	-10	-20	-7	18.24
18-HETE	319	261	-90	-10	-22	-9	18.15
19-HETE	319	231	-95	-10	-24	-11	17.85
20-HETE	319	245	-70	-10	-22	-17	17.97
5,6-EET	319	191	-65	-10	-16	-12	22.03
8,9-EET	319	155	-15	-10	-20	-5	22.06
11,12-EET	319	167	-40	-10	-22	-7	21.88
14,15-EET	319	219	-60	-10	-18	-7	21.09
5,6-DHET	337	145	-5	-10	-24	-27	17.68
8,9-DHET	337	127	-45	-10	-30	-13	17.44
11,12-DHET	337	167	-100	-10	-26	-17	17.22
14,15-DHET	337	207	-35	-10	-28	-9	16.98
PGD2	351	271	-50	-10	-23	-10	15.04
6-keto-PGF1a	369	163	-35	-10	-38	-15	13.05
15-d12,14-PGJ2	315	271	-55	-10	-22	-7	17.78
TxB2	369	169	-50	-10	-22	-10	14.17
PGE2	351	271	-50	-10	-23	-10	14.88
PGF2a	353	193	-50	-10	-35	-10	14.69
11(12)-EET-d11	330	167	-95	-10	-18	-9	21.88

Supplementary Table S2. A list of primer sets for real–time PCR.

Organisms	Name	Forward primer	Reverse primer
Mouse	*Gapdh*	5'-AGGTCGGTGTGAACGGATTTG-3'	5'-TGTAGACCATGTAGTTGAGGTCA-3'
	Rn18s	5'-GTAACCCGTTGAACCCCATT-3'	5'-CCATCCAATCGGTAGTAGCG-3'
	Srebf1	5'-CCGGCTATTCCGTGAACATC -3'	5'-GATATCCAAGGGCATATGAGAACT-3'
	Srebf2	5'-TGTGCGCTCTCGTTTTACTGA -3'	5'-GTATAGAAGACGGCCTTCACCAA-3'
	Mlxipl	5'-CGACACTCACCCACCTCTTC-3'	5'-TTGTTCAGCCGGATCTTGTC-3'
	Nr1h3	5'-AGGTACAACCCCGGCAGTGA-3'	5'-GCAGCTCATTCATGGCTCTGGA-3'
	Nr1h2	5'-GGTGCCAGGGTTCTTGCAGT-3'	5'-CGTGGTTGTAGCGTCTGGCT-3'
	Pparg	5'-CGCTGATGCACTGCCTATG-3'	5'-AAGTTGGTGGGCCAGAATG-3'
	Gpam	5'-AGGGCATGTTTGCCACCAGT-3'	5'-GGTTCAACTCCGCAGCCACT-3'
	Scd1	5'-GAGCCACAGAACTTACAAGGCACGG-3'	5'-TGTGAGGGTCGGCGTGTGTTTC-3'
	Elovl6	5'-TCAGCAAAGCACCCGAACTAGGTGA-3'	5'-ATGAACCAACCACCCCCAGCGA-3'
	Fasn	5'- TCCTGGAACGAGAACACGATCT-3'	5'- GAGACGTGTCACTCCTGGACTTG -3'
	Dgat1	5'-GGCCTGCCCCATGCGTGATTATTG-3'	5'-TGCCAGAGCAAACACGGAACCCA-3'
	Dgat2	5'-AACACGCCCAAGAAAGGTGGCAG-3'	5'-TGGTGGTCAGCAGGTTGTGTGTCTT-3'
	Cd36	5'-AGATGAATGGTTGAGACCCCGTGCC-3'	5'-ACCATCCACCAGTTGCTCCACACA-3'
	Acaca	5'-GCGATCTATCCGTCGGTGGT-3'	5'-TGTTGGGTCCTCCAGGCACT-3'
	Pklr	5'-GTGCCGCAGCCATCATTGTG-3'	5'-CAATGACAGCAGCCCGAGGT-3'
	Pck	5'-TCCCCAAAACTGGCCTCAGC-3'	5'-CCAGTGGCCCCATGCTGAAT-3'
	G6pc	5'-CATGGGCGCAGCAGGTGTAT-3'	5'-AGCTGCACAGCCCAGAATCC-3'
	Slc2a2	5'-TTCTTTGGTGGGTGGCTCGG-3'	5'-CAATGATGAGGGCGTGTGCC-3'
	Tnf	5'-AAAAGCAAGCAGCCAACCAGGCA-3'	5'-CACGTCGCGGATCATGCTTTCTGT-3'
	Il6	5'-TAGTCCTTCCTACCCCAATTTC-3'	5'-TTGGTCCTTAGCCACTCCTTC-3'
	Il10	5'-TGACTGGCATGAGGATCAGCAGGGG-3'	5'-TAGGAGCATGTGGCTCTGGCCGACT-3'
	Ccl2	5'-TGTCATGCTTCTGGGCCTGCTGT-3'	5'-AATGAGTAGCAGCAGGTGAGTGGGG-3'
	Adgre1	5'-TGGGGCTTTTGGCTGCTCCTCTT-3'	5'-CCCTGGAGAAGAGCTACGAACTGCC-3'
	Timp1	5'- ACAGCCTTCTGCAACTCGGACCT-3'	5'- GCGGCATTTCCCACAGCCTTGA-3'
	Timp2	5'- TCTTCAGCAGTGTGCGGGGTCT-3'	5'- GTGCATCTTGCCATCTCCTTCTGCC-3'
	Tgfb1	5'-GGAGCCTGGACACACAGTACAGCAA-3'	5'-CCACGTAGTAGACGATGGGCAGTGG-3'
	Tgfb2	5'-TGGCCGAGCAGCGGATTGAACT-3'	5'-TCACCCTCCGCTCTGGTTTTCACA-3'
	Col1a1	5'-TCAGCTTTGTGGACCTCCGGCT-3'	5'-ATACCTCGGGTTTCCACGTCTCACC-3'
	Acta2	5'-CCCTGGAGAAGAGCTACGAACTGCC-3'	5'-TGGTTTCGTGGATGCCCGCTGA-3'
	Mboat7	5'-CGCACCTTTCCGCTCCTACGTTTT-3'	5'-CGCAAGGCTGACTCCAAATAGCCC-3'
Human	*ACTB*	5'-CAAGGCCAACCGCGAGAAGATGAC-3'	5'-AGAGGCGTACAGGGATAGCACAGCC-3'
	SREBF1	5'-TGCTGGCTCACCTTTCCAGAGCA-3'	5'-CTCTGGCTTTGCCTTGCTGTCCTC-3'
	PPARG	5'-GCAAACCCCTATTCCATGCTG-3'	5'-TGGCATCTCTGTGTCAACCAT-3'
	MLXIPL	5'-TCACACGCCTCTTCGAGTGC-3'	5'-ATGGCGTTGTTCAGGCGGAT-3'
	FASN	5'-CTTCGGAGTCCACCCCAAGC-3'	5'-ACGCCAGTGTGTGTTCCTCG-3'
	ELVOL6	5'-CGGTGGTCGGCACCTAATGA-3'	5'-AGCACCAGTTCGAAGAGCACC-3'
	ACACA	5'-TCCTTGTCACCTGCTTCTGTTGGCT-3'	5'-TGTCTCGGCCCTGCTTTACTAGGTG-3'
	ACACB	5'-AGCGGGCCATCCGGTTTGTTGT-3'	5'-ACGGGGACGTAATGATCCGCCA-3'
	SCD	5'-AGGATGAGGGAAGCGAAGCAAGAGG-3	5'-CAGCCACTTTTTACCCCGAGCCAG-3'
	DGAT1	5'-GCCTTTCTGGTGTGGGTCTGGTGA-3'	5'-GCTGCCCAGGTTCCAAGTGAAGGA-3'
	DGAT2	5'-ACACACCCAAGAAAGGTGGCAGGA-3'	5'-TGGTGGTCAGCAGGTTGTGTGTCTT-3'
	PPARA	5'-TGGTGGACACGGAAAGCCCACT-3'	5'-GCTCGAAGCTGGTGAAAGCGTGT-3'
	CPT1A	5'-GCCATCCTGCTTTACAGGCGCAA-3'	5'-CTGAGCGGAGCAGAGTGGAATCGT-3'
	CPT2	5'-TCTTGGGGTGATGGTGTGGCAGT-3'	5'-TAGCTGGCTGGCTCTGTGGAGTGA-3'

BMJ Publishing Group Limited (BMJ) disclaims all liability and responsibility arising from any reliance placed on this supplemental material which has been supplied by the author(s)

Supplementary Table S3. A list of primers and probes for Quantitative PCR by the TaqMan System.

TaqMan System.

Name	Assay No.
IL1B	Hs01555410
IL6	Hs00174131
CCL2	Hs00234140
MMP9	Hs00957562
TIMP1	Hs00171558
TIMP2	Hs00234278
TGFB1	Hs00998133
TGFB2	Hs00234244
COL1A1	Hs00164004
ACTA2	Hs00426835
MBOAT7	Hs00383302
ACTB	Hs01060665

Supplementary Table S4. A list of sgRNA sequences for generating LPIAT1 KO cells.

Name	sgRNA sequences
MBOAT7	AGCTACAGCTACTGCTACGT
